# Estimating individualized treatment effects using an individual participant data meta-analysis

**DOI:** 10.1186/s12874-024-02202-9

**Published:** 2024-03-25

**Authors:** Florie Bouvier, Anna Chaimani, Etienne Peyrot, François Gueyffier, Guillaume Grenet, Raphaël Porcher

**Affiliations:** 1 Université Paris Cité and Université Sorbonne Paris Nord, Inserm, INRAE, Center for Research in Epidemiology and StatisticS (CRESS), Paris, France; 2 Cochrane France, Paris, France; 3 Laboratoire de Biométrie et Biologie Evolutive UMR 5558, CNRS, Université Lyon 1, Université de Lyon, Villeurbanne, France; 4 Centre d’Épidémiologie Clinique, AP-HP, Hôtel-Dieu, Paris, France

**Keywords:** Personalized medicine, Individualized treatment effects, Individual patient data, Meta-analysis

## Abstract

**Background:**

One key aspect of personalized medicine is to identify individuals who benefit from an intervention. Some approaches have been developed to estimate individualized treatment effects (ITE) with a single randomized control trial (RCT) or observational data, but they are often underpowered for the ITE estimation. Using individual participant data meta-analyses (IPD-MA) might solve this problem. Few studies have investigated how to develop risk prediction models with IPD-MA, and it remains unclear how to combine those methods with approaches used for ITE estimation. In this article, we compared different approaches using both simulated and real data with binary and time-to-event outcomes to estimate the individualized treatment effects from an IPD-MA in a one-stage approach.

**Methods:**

We compared five one-stage models: naive model (NA), random intercept (RI), stratified intercept (SI), rank-1 (R1), and fully stratified (FS), built with two different strategies, the S-learner and the T-learner constructed with a Monte Carlo simulation study in which we explored different scenarios with a binary or a time-to-event outcome. To evaluate the performance of the models, we used the *c*-statistic for benefit, the calibration of predictions, and the mean squared error. The different models were also used on the INDANA IPD-MA, comparing an anti-hypertensive treatment to no treatment or placebo ($N = 40\,237$, 836 events).

**Results:**

Simulation results showed that using the S-learner led to better ITE estimation performances for both binary and time-to-event outcomes. None of the risk models stand out and had significantly better results. For the INDANA dataset with a binary outcome, the naive and the random intercept models had the best performances.

**Conclusions:**

For the choice of the strategy, using interactions with treatment (the S-learner) is preferable. For the choice of the method, no approach is better than the other.

**Supplementary Information:**

The online version contains supplementary material available at 10.1186/s12874-024-02202-9.

## Background

Personalized (or stratified) medicine aims at tailoring a treatment strategy to the individual characteristics of each patient. One key aspect of personalized medicine is to identify individuals who benefit from an intervention. Different approaches exist, with a popular one being the estimation of the so-called individualized treatment effect (ITE). Shortly, the ITE on an additive scale is the predicted benefit under one treatment minus the predicted benefit under the other treatment, given a set of patients’ characteristics. It represents what treatment effect is expected for a patient with these characteristics. ITEs are generally estimated by building prediction models or by using machine learning methods such as random forests [1].

In practice, prediction models for ITE are often developed using data from a single randomized controlled trial (RCT) or observational data [2]. RCTs benefit from randomization but are often underpowered for such a task, which may lead to overfitting or the failure of capturing the effects of many relevant variables. A solution to that problem might be to use individual participant data meta-analyses (IPD-MA), which include larger numbers of patients and may also benefit from increased generalizability. Nevertheless, it is necessary to consider the variation between studies in such data to avoid bias. Previous studies have tackled the incorporation of heterogeneity when estimating the average treatment effect i.e. the average difference of the predicted risk between treatments, or have used IPD-MA to develop risk prediction models [3, 4]. However, it is unclear how to deal with heterogeneity in an IPD-MA while using approaches to estimate ITEs. Fisher et al. [5] and, more recently, Chalkou et al. [6] considered a framework to estimate the ITE in IPD-MA with a two-stage approach. More specifically, Chalkou et al. used a network meta-analysis with individual participant data to, first, estimate a prognostic model. Heterogeneity of treatment effects according to baseline risk predicted by this model was then considered using a two-stage approach with treatment by baseline risk interactions estimated within each trial. Seo et al. used one-stage meta-analytic approaches and focused on methods for selecting which treatment-covariate interactions to include in a model where study-specific intercepts and common effects factors were added; they concluded that shrinkage methods performed better than non-shrinkage methods [7].

In the context of a single study or RCT, a wide range of approaches have been proposed to estimate ITEs [8–12]. To our knowledge, how to adequately combine those with the approaches accounting for heterogeneity in IPD-MA has not been investigated. In this work, we considered two strategies called meta-learners, the S-learner and the T-learner [8].

In this study, we aimed to study the performance of strategies that estimate the ITE from an IPD-MA in a one-stage approach and methods focusing on taking into account the heterogeneity in baseline risks to understand which strategy and method should be used in practice. Different methods were compared using both simulated and real data with binary and time-to-event outcomes. We first present the different models and approaches compared in estimating ITEs. Next, we describe the Monte Carlo simulation study and its results, and the models are then applied to the data of the INDANA meta-analysis, a real individual patient data meta-analysis evaluating anti-hypertensive treatments [13]. We conclude with some discussion and paths for future research.

## Methods to estimate individualized treatment effects

In this section, we described the different approaches we compared to estimate the ITE from an IPD-MA accounting for the clustering of patients within trials. We first explain the two approaches used to obtain ITEs from risk prediction models and then the different approaches to develop risk prediction models in an IPD-MA we considered.

### ITE estimation

Let us consider a binary outcome without loss of generality. The case of time-to-event outcomes, which is similar in essence, is described in Supplementary Material S1. The ITE, which is the difference in predicted benefits of two treatments given a set of patients’ characteristics, is estimated as:

$$\begin{aligned} \hat{\tau }(x) = \hat{\mu }(x,1) - \hat{\mu }(x,0). \end{aligned}$$

where $\hat{\mu }(x,z)$, $z \in \{0,1\}$ represents the predicted mean outcome under treatment *z* for an individual with covariates *x*.

To estimate the ITE $\tau$ many methods exist. In this project, two meta-learners were used, the S-learner and the T-learner, which decompose the estimation of the ITE into sub-regression problems [8]. The meta-learners can be implemented with various prediction techniques such as regression or random forests for instance. In this work, we decided to use regression since methods to handle the heterogeneity in an IPD-MA have been developed with regression in previous works [3, 4].

The S-learner estimates the ITE using a single regression model, where interactions between the indicator variable for the treatment and relevant covariates are introduced.

Considering for instance a logistic regression model, the S-learner consists in estimating the following model:

$$\begin{aligned} \text {logit} \mu (x,z) = \alpha + \theta ' x + \gamma z + \eta ' xz. \end{aligned}$$

From this, we derive for all individuals:

$$\begin{aligned} \hat{\mu }(x,1) = \text {expit}(\hat{\alpha } + \hat{\theta '} x + \hat{\gamma } + \hat{\eta '} x), \end{aligned}$$

and

$$\begin{aligned} \hat{\mu }(x,0) = \text {expit}(\hat{\alpha } + \hat{\theta '} x). \end{aligned}$$

Different approaches to obtain estimates of $\alpha , \theta , \gamma , \eta$ are described in the next subsection.

The T-learner estimates the ITE using two separate regression models, one built using data from the treatment group and one built using data from the control group. The two following models:

$$\begin{aligned} \text {logit} \mu (x,0) = \alpha ^{0} + \theta '^{0} x, \end{aligned}$$

for individuals with $z = 0$ and

$$\begin{aligned} \text {logit} \mu (x,1) = \alpha ^{1} + \theta '^{1} x, \end{aligned}$$

for individuals with $z = 1$, are fitted and $\hat{\tau }$ is obtained from $\hat{\mu }(x,1) = \text {expit}(\hat{\alpha } + \hat{\theta '}^1 x)$ and $\hat{\mu }(x,0) = \text {expit}(\hat{\alpha } + \hat{\theta '}^0 x)$ for all individuals in the meta-analysis.

The S-learner algorithm may reduce overfitting compared to the T-learner algorithm as it can adjust the number of interactions included in the model and thus can reduce the number of estimates. However, since IPD-MA is used in this work, the potential overfitting of the T-learner might be reduced due to a larger sample size.

In our case, we want to obtain $\hat{\mu }(x,z)$ using data from an IPD-MA. Several approaches exist to estimate this quantity while accounting for the potential heterogeneity that may arise in a meta-analysis. These approaches are detailed in the next subsection.

### Risk prediction models in IPD-MA

Let us consider an IPD-MA where data from individual patients from *J* randomized controlled trials are available, and the outcome of interest is binary. Different methods to develop a single risk prediction model using IPD-MA have been proposed [3, 4]. Four of them were compared in this work and a naive model, that ignores any heterogeneity which may occur between the different studies included in the meta-analysis, was added to the comparison.

Let $x_{ij} = (x_{ij1}, \ldots ,x_{ijN})$ be a vector of covariate values for subject $i \in (1, \ldots , N_j)$ in study $j \in (1,\ldots ,J)$. For the purpose of describing the different approaches, we do not differentiate the treatment indicator from other covariates and do not specify interactions between covariates, they could be incorporated in the definition of $x_{ij}$. We considered the following five models:

- Naive model (NA): A first approach considers that all data comes from a single population, and therefore assumes that there is no heterogeneity. In this model, a common intercept and common predictor effects are included. This naive approach can lead to bias when heterogeneity is actually present. The model can be expressed as:
  1 $$\begin{aligned} \text {logit}(p_{ij}|x_{ij}) = \alpha + \theta ' x_{ij} \end{aligned}$$
  where $p_{ij}$refers to the probability of subject *i* in trial *j* to develop the outcome. When individual predictions are made to estimate the ITE at a covariate level *x*, these predictions are obtained by $\hat{\mu }(x) = \text {expit}(\hat{\alpha }+\hat{\theta '}x)$, where $\hat{\alpha }$ and $\hat{\theta }$ are the Maximum likelihood estimators of $\alpha$ and $\theta$ respectively, in the model (1).
- Random intercept model (RI): A second approach is to assume that the heterogeneity in the IPD-MA occurs only on the baseline risk i.e. the intercept varies between studies, but the effects of all predictors are the same in each study. In this model, we consider a random study effect to model the distribution of the intercept across studies. The underlying model can be written as:
  2 $$\begin{aligned} \text {logit}(p_{ij}| x_{ij}, \alpha _j) = \alpha _j + \theta ' x_{ij} \end{aligned}$$
  with $\alpha _j \sim \mathcal {N}(\alpha , \tau _{\alpha }^2)$. The individual predictions are obtained by $\hat{\mu }(x) = \text {expit}(\hat{\alpha }+\hat{\theta '}x)$. Estimators of $\alpha$ and $\theta$ are obtained via maximum likelihood which is approximated with the adaptive Gauss-Hermite quadrature.
- Stratified intercept model (SI): A third approach is to include a different intercept for each study, as a fixed effect. With a binary outcome:
  3 $$\begin{aligned} \text {logit}(p_{ij}|x_{ij}, \alpha _j) =\sum _{m=1}^J \alpha _m I (m=j) + \theta ' x_{ij} \end{aligned}$$
  where $I(\cdot )$ denotes the indicator function. To derive the individual predictions as $\hat{\mu }(x) = \text {expit}(\hat{\alpha }+\hat{\theta '}x)$, the estimator of $\theta$ is obtained via maximum likelihood. To obtain a single $\hat{\alpha }$ , we used a random-effects meta-analysis of the $\alpha _m$, with inverse variance weighting, as suggested by Debray et al. and Royston et al. [3, 14]. The choice of a random-effects meta-analysis was based on considering that using separate intercepts for each study implied that some heterogeneity would be expected.
- Fully stratified model (FS): A fourth approach is to consider that there is heterogeneity across studies on both the baseline risks and the predictors’ effects. In that case, we calculate different intercept and predictor effects for each trial included in the meta-analysis. With a binary outcome:
  4 $$\begin{aligned} \text {logit}(p_{ij}|x_{ij}, \alpha _j, \theta _j) =\sum _{m=1}^J (\alpha _m I (m=j) + \theta '_m I (m=j) x_{ij}) \end{aligned}$$
  where $\alpha _m$ and $\theta _m$, for $m = 1,..,J$, are real valued parameters to be estimated. This is equivalent to fitting a separate model in each study included in the meta-analysis. The individual predictions are then obtained as $\hat{\mu }(x) = \text {expit}(\hat{\alpha }+\hat{\theta '}x)$, where a single intercept estimate $\hat{\alpha }$ and single predictor estimates$\hat{\theta }$ are obtained with a random-effects multivariate meta-analysis.
  $$\begin{aligned} \left( \begin{array}{c} \alpha _m\\ \theta _m \end{array}\right) \sim \text {MVN}\left( \left( \begin{array}{c} \alpha \\ \theta \end{array}\right) ,V\right) \end{aligned}$$
  where *V* is the between-study covariance matrix of the intercept and the predictor effects.
- Rank-1 model (R1): A final approach considers that the linear predictors share a common direction in the covariate space but that the size of their effects might be systematically different [15]. This model can be thought of as an intermediate between the common effect models and the fully stratified model. In this setting, the study-specific effects can vary in a proportional way, modeled by a random effect $\phi$. With a binary outcome:
  5 $$\begin{aligned} \text {logit}(p_{ij}|x_{ij}, \alpha _j, \phi _j) = \alpha _j + \phi _j \theta x_{ij} \end{aligned}$$
  with $$\alpha _j \sim \mathcal {N}(\alpha , \tau _{\alpha }^2)$$, $$\phi _j \sim \mathcal {N}(1, \tau _{\phi }^2)$$
  With the rank-1 model, the individual predictions are acquired by $\hat{\mu }(x) = \text {expit}(\hat{\alpha }+\hat{\theta '}x)$, where both estimators are directly obtained as in the random intercept model.

The risk models using a time-to-event outcome are described in Section 1 of the Supplementary Material.

### Model validation

Internal-external cross-validation (IECV) was used to validate the models. In the IECV, the model is constructed with $J-1$ studies and validated with the remaining study for each permutation of $J-1$ studies. To account for the heterogeneity in baseline risk, the model is re-calibrated in the test datasets. We first estimate the intercept with the different risk prediction models presented previously. Recalibration is then performed by estimating a regression model with the linear predictors $\hat{\theta }x$ of the original model as an offset i.e. the regression parameter is forced to be one. These steps are performed for all models except for the naive model which ignores all potential heterogeneity. To assess the models’ performance, discrimination and calibration were considered. We also calculated the mean squared error.

To assess the discrimination, which is the ability of the model to distinguish between individuals who benefit and individuals who do not benefit from taking the treatment, the c-statistic for benefit proposed by van Klaveren et al. [16] was used. Since the individual benefit, i.e. obtaining a more favorable outcome when taking the treatment than when not taking it cannot be observed, van Klaveren et al. used pairs of individuals, one in each treatment group, with close predicted ITE to approach the individual benefit. The c-statistic for benefit is the extension of the c-statistic for individualized treatment effects. The c-statistic for benefit is defined as the probability that from two randomly chosen matched pairs (*p*1, *p*2) with unequal estimated benefit, the pair with greater estimated benefit also has a higher predicted probability, where the estimated benefit refers to the difference in outcomes between two patients with the same predicted benefit but with different treatment assignments. To create the pairs, a patient in the control group is matched to one in the treatment group with a similar predicted treatment benefit. Higher values of the c-statistic for benefit are better. The c-statistic for benefit can be expressed as:

$$\begin{aligned} C_{\text {for-benefit}} = P\Big (\hat{\tau }(x_{p1})>\hat{\tau }(x_{p2}) \,|\, \tau (x_{p1})>\tau (x_{p2})\Big ) \end{aligned}$$

where $\tau (x_{p1})$ and $\tau (x_{p2})$ represent the observed benefits of pairs *p*1 and *p*2 and where $\hat{\tau }(x_{p1})$ and $\hat{\tau }(x_{p2})$ represent the predicted benefits of pairs *p*1 and *p*2 respectively.

For the calibration, the agreement between the observed and the predicted benefit, was assessed by extracting the intercept and the slope of the regression line. An intercept close to 0 and a slope close to 1 indicate a good calibration. Calibration curves were also plotted when the methods were applied to the INDANA dataset. The predictions were divided into five bins; to make sure to include individuals who were allocated to the treatment and individuals who were allocated to the control. In each bin, the mean of the predicted benefit was compared to the observed benefit.

### Addressing aggregation bias

An issue related to the one-stage approach is the way treatment-covariate interactions are included. Indeed, if the model is not correctly specified, it can lead to aggregation bias which occurs when using the information across studies modifies the interactions’ estimates obtained when using only within-study information. In order to avoid aggregation bias, only within-trial interaction should be used to estimate the treatment-covariate interactions. To make sure only within-trial information is used, a solution to distinguish within- and across-trial information has been described in Riley et al. [17]. This method consists in centering the covariates to their study-specific mean and adding the covariates’ mean as an adjustment term that explains between-study heterogeneity. Since within- and across-trial information are now uncorrelated, we are able to solely use within-trial information. After conducting some simulations (details are given in Section 2 of the Additional file 1) in which we compared the estimates obtained with the models described in the previous section with and without the aforementioned method, we concluded that not centering variables to their study-specific mean and not including a covariate-mean interaction term did not lead to aggregation bias with the proposed models since the estimates obtained were similar. In their paper, Belias et al. find that using this method leads to very small differences [18]. Therefore, we decided to evaluate the performance of the different models without including the method.

### Implementation

All the analyses were performed in R version 4.1.2. The random intercept and the stratified intercept models were developed using glmer from the lme4 package for binary outcomes and using coxme from the coxme package for time-to-event outcomes. For the rank-1 models, we used rrvglm in the VGAM package and coxme in coxme. Finally, the fully stratified model was developed using glm and coxph from survival.

## Monte Carlo simulation study

### Setting

The performance of the models and meta-learners was evaluated in a simulation study. We considered 24 scenarios in which we changed the number of covariates, the number of patients in each trial, and the type of outcome. The scenarios are briefly described below, and more details are given in Section 3 of Additional file 1. We simulated 1000 IPD-MAs composed of 7 trials for each scenario. All the continuous covariates were drawn from a normal distribution and all the binary covariates were drawn from a Bernoulli distribution. For individual *i* in study *j*, the treatment allocation $t_{ij}$ was sampled from a Bernoulli distribution of parameter 0.5, the binary outcome $y_{ij}$ was generated from a Bernoulli distribution of parameter $p_{ij}$, where $\text {logit}(p_{ij}) = \alpha _j + \theta _j x_{ij} + \gamma _j t_{ij}$ and the time-to-event outcome was generated from a Weibull distribution $f(x;k,b) = bkx_{ij}^{k-1}\exp {(-bx^{k})}$, where *k* represents the shape parameter and *b* the scale parameter. We chose $k = 1.15$ i.e. the failure rate increases over time and $b = \frac{a}{\exp {(\theta _j x_{ij})}^{k}}$, with $a=50$ to obtain a stretch distribution.

In 12 scenarios, data was generated with a common treatment effect (all $\gamma _j=\gamma$), whereas in the other 12, we included some variation in the predictor effects.

In scenarios 1 to 3, we considered IPD-MAs with a total number of patients equal to 2800, 1400 and 700 respectively (for simplicity, trials were of identical sample size) composed of 3 covariates, 3 treatment-covariate interactions and a binary outcome. Among the covariates, one of them was binary and the other two were continuous.

In scenarios 4 to 6, we computed IPD-MAs with a total number of patients equal to 2800, 1400 and 700 (trials were of identical sample size) composed of 9 covariates (6 binary and 3 continuous) and 4 treatment-covariate interactions.

Scenarios 7 to 12 had the same configuration as scenarios 1 to 6 but the predictor effects varied according to the trial for some variables.

Scenarios 13 to 18 had the same configuration as scenarios 1 to 6 and scenarios 19 to 24 were similar to scenarios 7 to 12 but instead of a binary outcome, we used a time-to-event outcome.

A summary of all scenarios can be found in Supplementary Table 7 of Additional file 1.

We also tackled the impact of variables’ selection on the performance of the approaches. We performed variables’ selection using a Group lasso [19] for scenarios 4 to 6 and 10 to 12 with the stratified intercept model. The results can be found in Supplementary Material S3.

### Results

Results of scenarios 1 to 6 and 13 to 18 are available in Section 4 of Additional file 1. If outliers were found in the results, they were removed from the analysis.

**Fig. 1:**
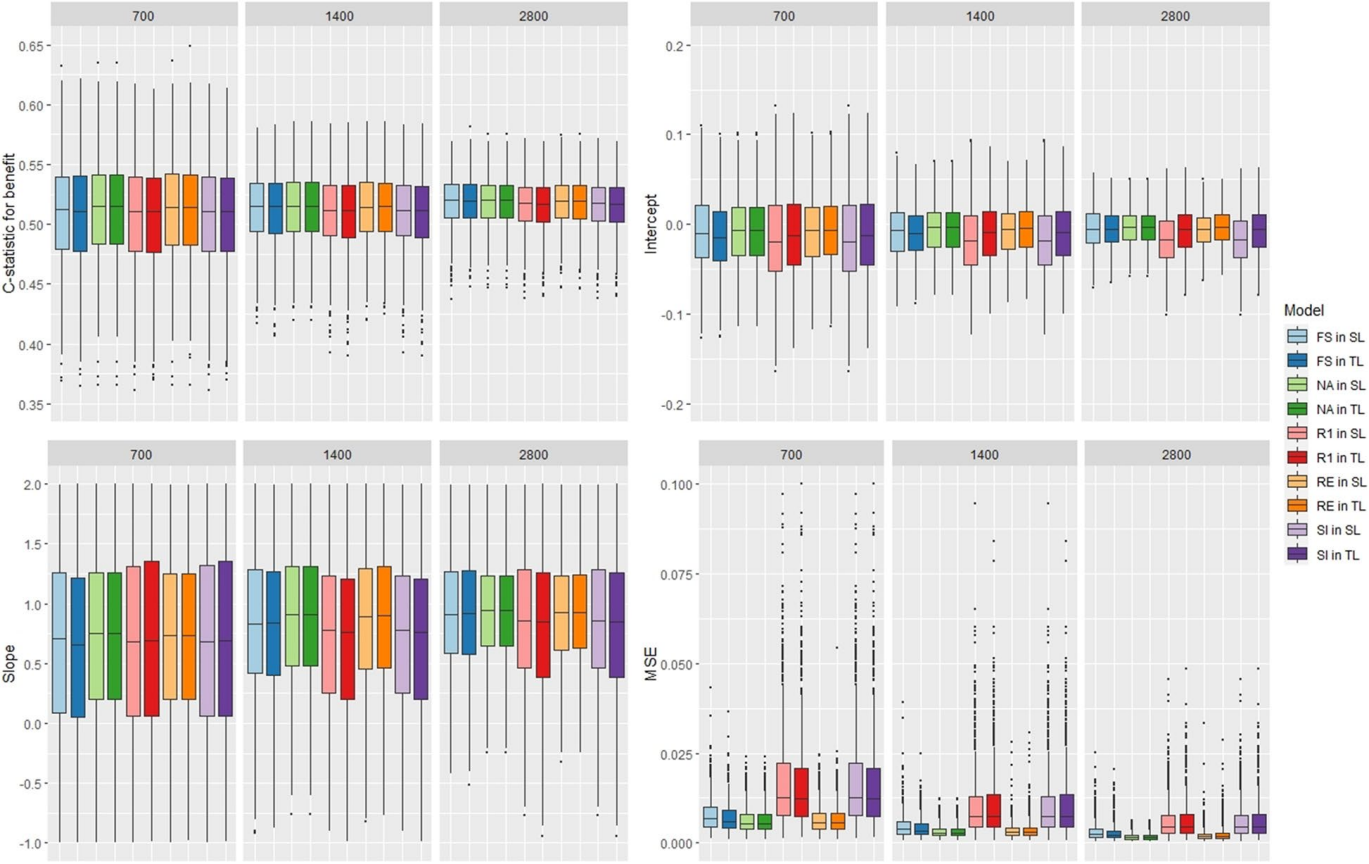
Boxplot of the models’ performance with 3 covariates, a binary outcome, and variation in the predictor effects

**Fig. 2:**
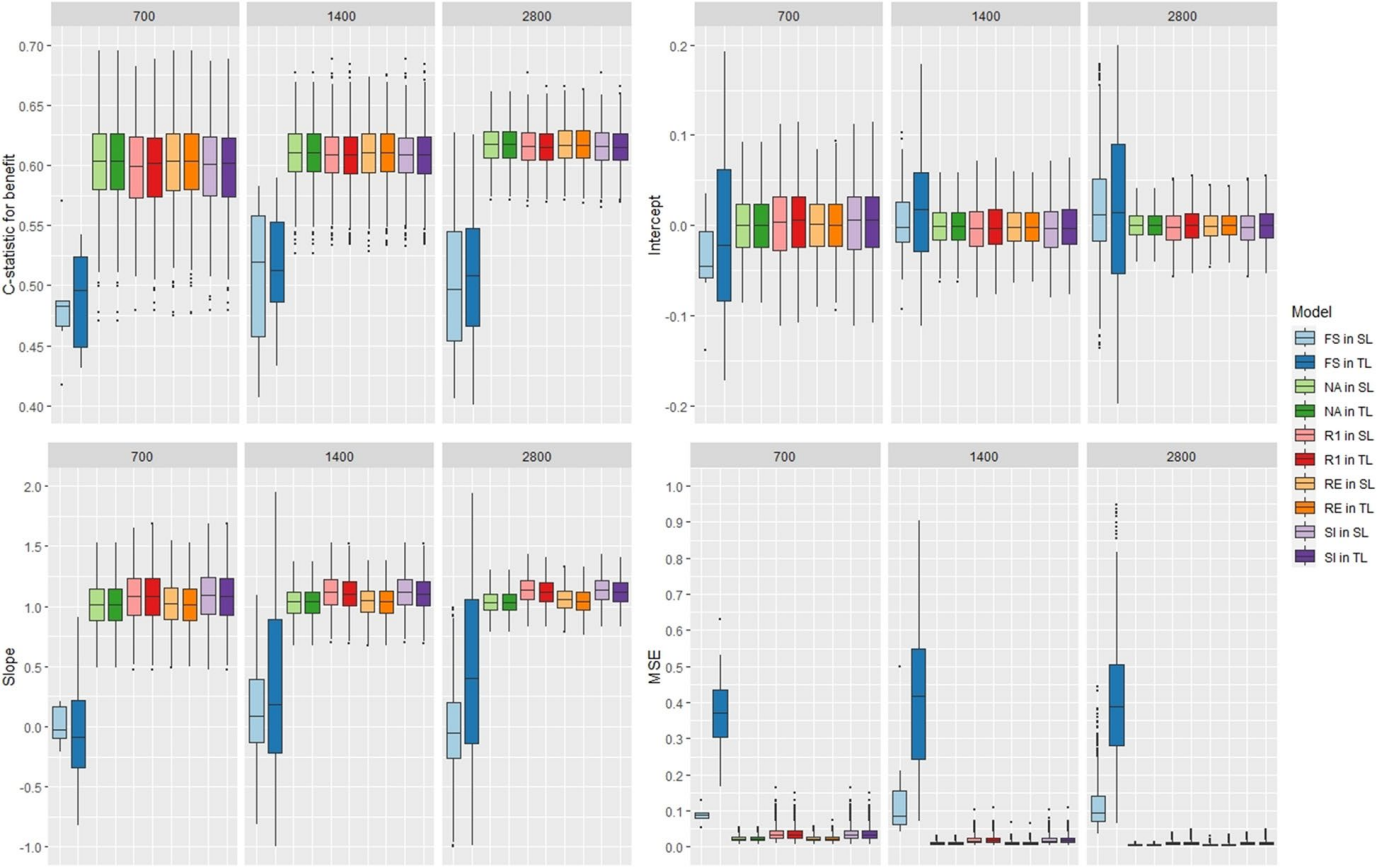
Boxplot of the models’ performance with 10 covariates, a binary outcome, and variation in the predictor effects

With 3 covariates, all methods had a nearly equal performance, in terms of discrimination and calibration, with both meta-learners (Fig. 1). The mean c-statistic for benefit values were around 0.52 for all models, and although van Klaveren stated that it was difficult to obtain values over 0.6 [16], it still indicates poor discrimination. The calibration was mediocre, the intercept values were close to 0 but the slope values were not close to 1. The rank-1 and the stratified intercept models had higher MSE values and than the other model. With 9 covariates, the fully stratified under-performed the other models with lower discrimination and calibration as well as higher MSE values. The poor performance of FS might be due to overfitting, different intercept and predictor effects are included in the model for all studies (Fig. 2). The other models performed similarly, they had a good calibration with intercept values and slope values close to 0 and 1, respectively, and acceptable discrimination with c-statistic for benefit values around 0.6. Using the S-learner or the T-learner led to equivalent performances for the NA, RI, SI, and R1 methods. However, for the fully stratified method, it was preferable to use the S-learner approach to obtain better results and lower MSE values. Changing the size of the IPD-MA did not impact the results. When a binary outcome is used and the model includes few covariates, we recommend using the naive, random intercept or fully stratified models which have lower MSE. When the model includes more covariates, we recommend avoiding using the fully stratified model and favoring the other methods. Despite including some heterogeneity in the predictor effects between studies, the naive model, which ignores any potential heterogeneity, did not perform worse than the other methods. The naive model might underestimate some effects and overestimate others, thus leading to a similar performance to the other methods’ performances. Similar conclusions were reached in scenarios without variation in the predictor effects (results in Section 4 of Additional file 1).

**Fig. 3:**
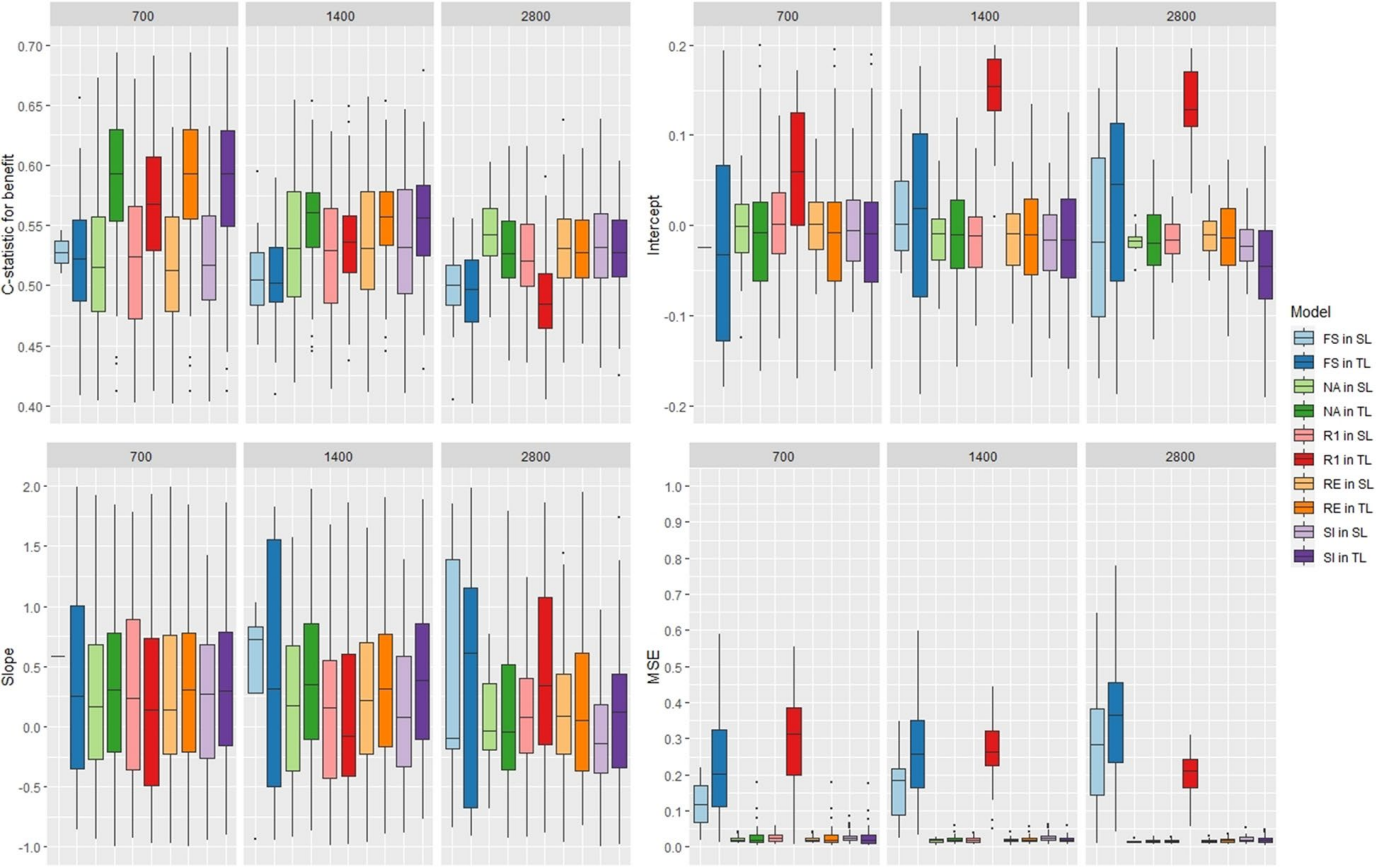
Boxplot of the models’ performance with 3 covariates, a time-to-event outcome, and variation in the predictor effects

When a time-to-event outcome was used with 3 covariates, we noticed that using the T-learner led to slightly better discrimination results for all methods, whereas using the S-learner led to better calibration results (Fig. 3). Slope values far from 1 indicating a poor calibration. Higher MSE values were obtained for the fully stratified model and for the rank-1 model when the T-learner was used. The NA, RI, and SI methods’ results were similar.

**Fig. 4:**
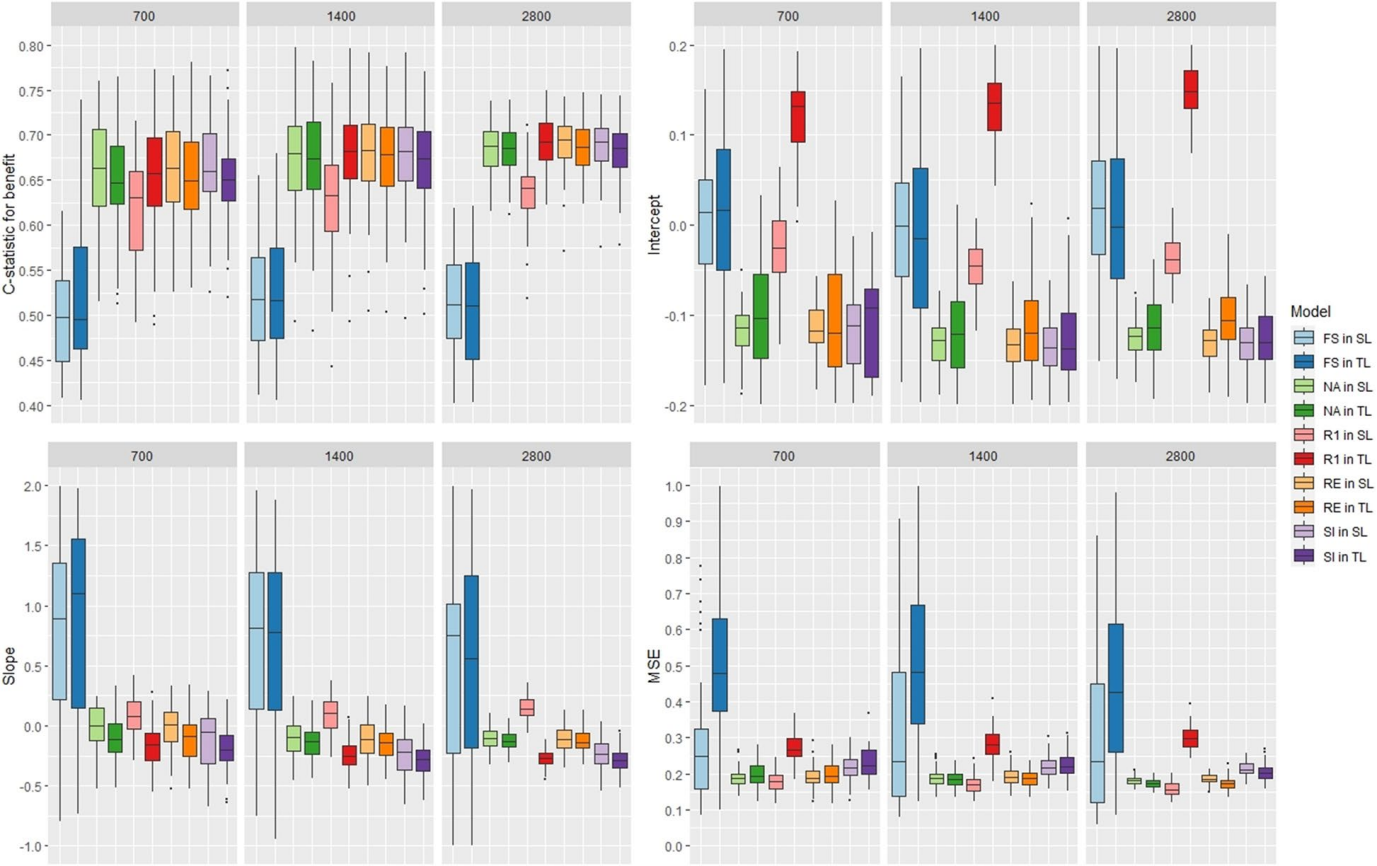
Boxplot of the models’ performance with 10 covariates, a time-to-event outcome, and variation in the predictor effects

With 9 covariates, using FS led to better calibration results but led to worse discrimination (Fig. 4). The other methods produced analogous discrimination results and had mean c-statistic for benefit values above 0.65. FS had the higher MSE. Overall, using the S-learner led to more stable results and led to lower MSE values.

When a time-to-event outcome is used, we recommend choosing the S-learner approach to estimate ITEs. No methods outperformed the others but with several covariates, the fully stratified model had the best calibration. Similar conclusions were obtained when variation in the predictor effects was not included (results in Section 4 of Additional file 1).

In scenarios where variation in predictor effects was included across studies, the rank-1 and the fully stratified models, which are the models that capture more heterogeneity, did not stand out from the other models and did not lead to better ITE estimation. The fully stratified, which estimates separate intercept and predictor effects for each study included in the meta-analysis, is prone to overfitting. This overfitting was seen in our results, particularly in scenarios with more covariates. The rank-1 model allows the predictor effects to vary in a proportional way, which might not capture well the type of heterogeneity we considered in the various scenarios (see Supplementary Material S3). In a scenario where heterogeneity was generated in a proportional way, the rank-1 model performed better (Supplementary Material S4).”

**Fig. 5:**
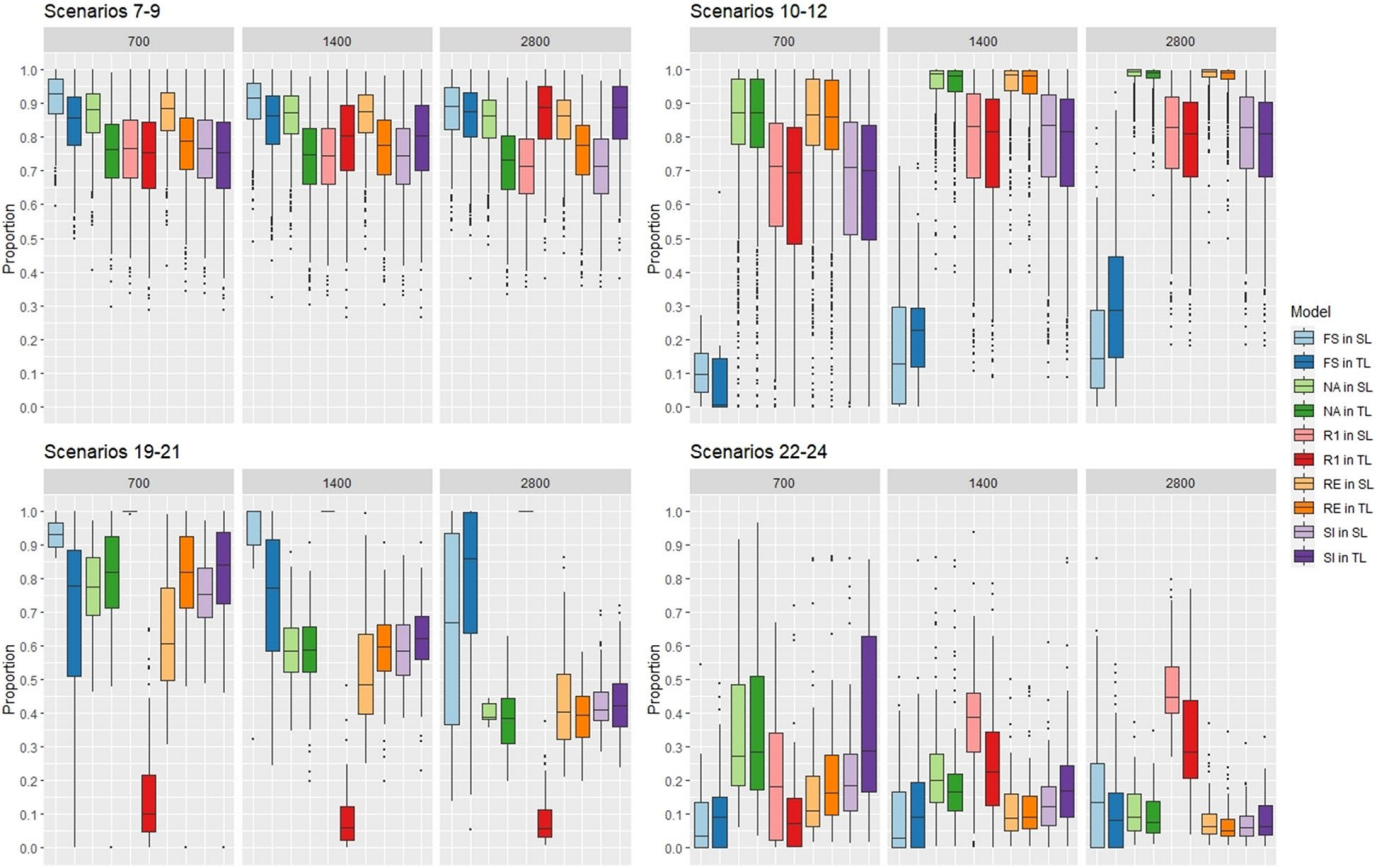
Number of times the true ITEs was in the prediction intervals of each model

To further investigate the performance of the methods, we computed prediction intervals around the predicted ITE of all individuals for each method and calculated the number of times the true ITE was included in the interval (Fig. 5). The predicted ITEs correspond to what would be expected as a treatment effect for an individual with characteristics $x_i$, had they been assigned to an average trial. In scenarios 7 to 9, FS’s prediction intervals were the ones that included the true ITE the most. In scenarios with 9 covariates and a binary outcome (scenarios 10 to 12), the true ITE was more often in the intervals of NA and RI. With a time-to-event, the prediction intervals of FS and R1 with the S-learner included the true ITE more frequently when 3 covariates were used. With 9 covariates, R1’s prediction models captured the true ITE more often. Overall, the two methods that included more heterogeneity were the ones that captured the most the true ITE in their prediction intervals.

## Illustration on real data

### INDANA IPD-MA

To illustrate the different approaches, we used data from the individual data analysis of antihypertensive intervention trials (INDANA) IPD-MA to evaluate the models [20]. This IPD-MA is composed of 9 randomized controlled trials comparing an antihypertensive treatment versus no treatment or a placebo, but given the large disparity between trials, notably for the variable age (See Supplementary Fig. 8, Additional file 1), we decided to compare the different methods on 4 of them for which the median age was under 60 years old. The outcome used in this project was death. The dataset was composed of 40 237 observations and 836 deaths. After comparing the calibration obtained with different combinations of variables, we decided to include the following variables in the final models: age, sex, systolic blood pressure (SBP), serum creatinine and treatment group (Table 1). Since some values were missing, we replaced them using a simple run of a multiple imputation procedure [21]. Considering that the dataset was only used for illustration, we considered that a single imputed dataset would be sufficient. For clinical research, it would be recommended to use several imputed datasets and pool the results [22]. Proper guidance for estimating ITE is lacking but could be adapted from techniques used for building risk prediction models [23, 24].

**Table 1:** Description of the predictors in each trial of the INDANA IPD-MA. The dataset with imputed missing data we analyzed is presented

Variable	ANBP	MRFIT	HDFP	MRC1
Age, mean (SD) years	50.1 (9.0)	46.9 (5.9)	50.8 (9.8)	52.1 (7.5)
Male, no. (%)	2475 (63.0)	8012 (100.0)	5910 (54.0)	9048 (52.1)
SBP, mean (SD) mmHg	154.3 (19.1)	141.1 (14.4)	158.8 (22.8)	161.6 (17.1)
Serum creatinine, mean (SD) $\mu$mol/l	87.2 (21.6)	98.0 (13.4)	94.1 (23.2)	84.8 (21.1)
Antihypertensive treatment arm, no. (%)	1988 (50.6)	4019 (50.2)	5485 (50.1)	8700 (50.1)

### Results

**Fig. 6:**
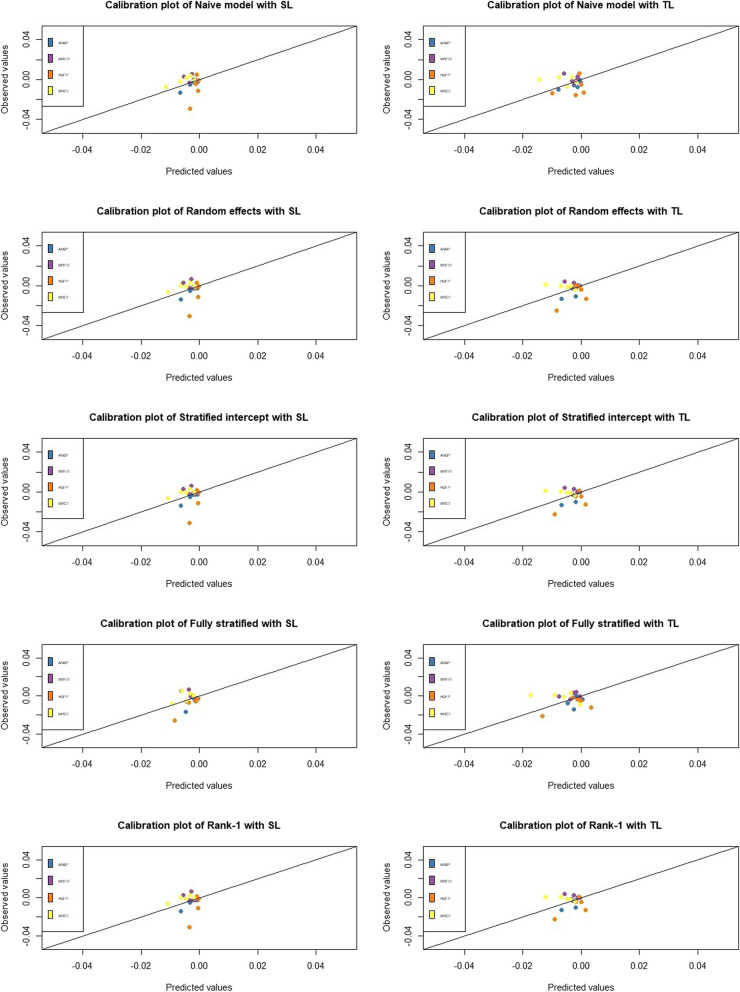
Calibration plots of the models built with S-learner (left) and T-learner (right) using INDANA with a binary outcome

Considering death as a binary outcome, a higher c-statistic for benefit was obtained with the S-learner rather than the T-learner, whatever method to handle heterogeneity was used (Table 2). No significant difference was found between the five methods, with c-statistic for benefit values close to 0.5. Even recalling that van Klaveren et al. mentioned it was usual to observe a c-statistic for benefit under 0.6, our results still showed a limited discrimination for the treatment effects [16]. Despite its large sample size (40 237 observations), the dataset only contained 836 events which could also explain why it was difficult to obtain models that discriminated well. In terms of calibration, the median intercept value was close to 0 for every model, with slightly better results when the S-learner was used. With the S-learner, the naive method had a slightly better median slope and the fully stratified method gave the values further from 1. With the T-learner, the RI method had a median slope closer to 1. The SI and R1 methods gave identical median slope values with both approaches. In general, median slope values were not close to 1 which we can visualize in Fig. 6 showing that most points are not close to the diagonal. The MSE values were close to 0 and comparable for every method whatever approach was used. The naive model and the random intercept method built with the S-learner produced the best performances with the INDANA dataset.

**Table 2:** Median results using INDANA with a binary outcome

	S-learner					T-learner				
	NA	RI	SI	R1	FS	NA	RI	SI	R1	FS
C-stat	0.530	0.530	0.530	0.530	0.530	0.507	0.524	0.524	0.524	0.518
Intercept	0.001	0.001	0.002	0.002	0.001	-0.003	-0.003	-0.003	-0.003	-0.003
Slope	1.433	1.453	1.460	1.460	1.961	0.268	0.727	0. 569	0. 569	0.596
MSE	0.000	0.000	0.000	0.000	0.000	0.000	0.000	0.000	0.000	0.000

The usefulness of adopting a personalized strategy with the INDANA dataset was assessed with three different metrics(Table 3). The individualized treatment rules developed with all methods were compared to a rule treating everyone and to a rule treating no one. The PAPE, which compares the ITR with a treatment rule that randomly treats the same proportion of patients, was also computed [25].

Results showed that there was almost no benefit of using a personalizing strategy with INDANA. PAPE were all around 0 indicating the ITRs did not improve the outcome compared to a rule that randomly treats the same proportion of patients. Similar results were obtained when comparing the ITRs to a rule that treats everyone or to a rule that treats no one. All methods performed similarly with both meta-learners. The limited gain in personalization might be due to the distribution of the treatment effects. Sufficient heterogeneity of treatment effects is needed to develop useful individualized treatment rules.

**Table 3:** Metrics to assess the usefulness of personalization on the INDANA dataset

	S-learner					T-learner				
	NA	RI	SI	R1	FS	NA	RI	SI	R1	FS
PAPE	0	0	0	0	0	0.001	0	0.001	0.001	0.001
$V(r)-E(Y(0))$	-0.003	-0.003	-0.003	-0.003	-0.003	-0.002	-0.002	-0.002	-0.002	-0.002
$V(r)-E(Y(1))$	0	0	0	0	0	0.001	0.001	0.001	0.001	0.001

**Fig. 7:**
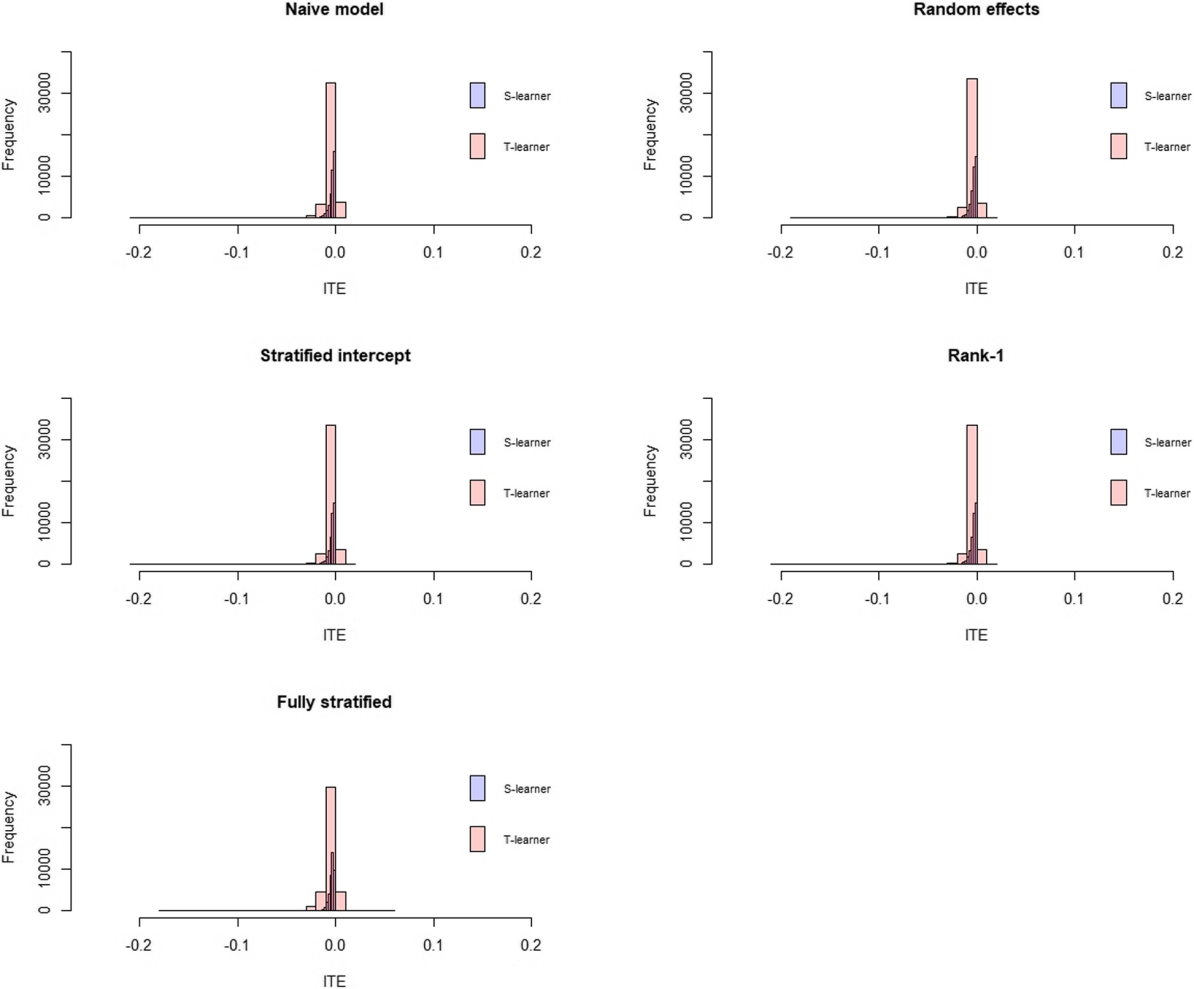
ITE distribution of the models with the S-learner (blue) and the T-learner (red)

The distributions of the individualized treatment effects estimated with the different methods were comparable when the same approach, the S-learner or the T-learner, was used (Fig. 7). With the S-learner, all the ITEs were negative. All the ITE estimates were close to 0 which explains the fact that it was difficult to discriminate individuals benefiting from individuals not benefiting from taking the treatment and might indicate a very small treatment effect.

When considering the performance of the methods and the approaches on the train dataset, the discrimination was still low and the calibration improved a bit, especially for the SI and R1 methods (Supplementary Material S5). The c-statistic for benefit values remained close to 0.5 and the ITEs estimated were close to 0 which explains the low discrimination. The fact that the performance did not drastically increase on the train dataset might indicate that the disparity between the trials was too high for them to be meta-analyzed.

## Discussion

This paper compared different approaches to estimate individualized treatment effects in an IPD meta-analysis. Using Monte Carlo simulations, the performance of those approaches was compared in terms of calibration and discrimination of the ITE. Eight approaches were considered, combining two strategies for model building (meta-learners), one where interactions between treatment and covariates are added in a regression model (S-learner), and one where two different regression models are fitted for each treatment group (T-learner), with five methods to handle the heterogeneity from the meta-analytic design: naive, random intercept, stratified intercept, fully stratified and reduced rank (rank-1) models. Both binary and time-to-event outcomes were studied. The methods were illustrated in a clinical example.

In the settings we considered, and with binary outcomes, using interactions with treatment (S-learner) or two different models (T-learner) had little impact on the model performance. With a binary outcome, we recommend avoiding using the fully stratified model when several covariates are included, as it is prone to overfitting. For time-to-event data, results were better when the S-learner approach was used but no methods stand out and outperformed the others. Additionally, considering variable selection did not change the performance of the algorithms. The rank-1 and the fully stratified models that include more heterogeneity were the methods that captured more of the uncertainty around the ITE prediction, and their prediction intervals included the true ITE more often than the prediction intervals of the other methods.

In this paper, the ITE was estimated using a one-stage approach. Estimation of the ITE can also be done with a two-stage approach. In a two-stage approach, Fisher et al. [5] advised only considering within-trial interaction i.e. calculating the difference of predicted outcomes in each trial and then comparing the results between trials. Chalkou et al. [6], who also used a two-stage approach to estimate the ITE with IPD-MA within a NMA framework, found that using a pre-specified model (a model with previously identified prognostic factors) rather than a LASSO model yielded better results.

To our knowledge, only one other study investigated the performance of ITE estimation in IPD-MA in a one-stage approach [7] but focusing on variable selection in models with treatment-by-predictor interactions (S-learner) only. In our work, we also considered the more flexible T-learner approach, whose performance was close to the S-learner when a binary outcome was considered. Since the T-learner may allow non-parametric interactions between treatment and predictors, our results for binary outcomes, slightly differ from recent reports suggesting that models with effect interactions were prone to over-fitting [26]. This may be explained by the use of IPD-MA. Indeed, we had hypothesized that in IPD-MA, where the number of predictors is often limited and the sample size is large, the issues related to over-fitting could be less important. Both simulations and analysis of a real dataset confirmed this hypothesis. We also focused more on the performance of ITE estimation in terms of calibration and discrimination (c-statistic for benefit) of the treatment effect estimate, i.e., on the models’ ability to correctly estimate the ITE and separate between individuals benefiting from the treatment and others. Discrimination and calibration are both important to guide treatment decisions.

Steyerberg et al. [4], who compared three of the methods presented in this paper (naive, random intercept, and rank-1) for risk prediction models using an IPD-MA, concluded that rank-1 was the most appropriate method. Here, to estimate the ITE with an IPD-MA, rank-1 did not perform well, especially with a time-to-event outcome.

When deriving predictions for individuals from a new study, we chose to compute a single intercept by performing a meta-analytic approach. Other methods can be employed such as selecting the intercept from the most similar study or estimating the intercept using the outcome prevalence [3]. In our case, without any specific target population in the simulation study and real data analysis, we considered that pooling the intercepts with a meta-analysis would be adequate.

In this work, we decided to use regression to estimate the ITEs. One limitation of regression models is the risk of model misspecification and its impact on estimated ITEs. Within the framework of meta-learners, it is possible to use non-parametric machine-learning approaches such as random forests. Moreover, more robust methods exist to estimate ITEs, such as the R-learner and the DR-learner [9, 27]. Robust approaches for creating individualized treatment rules (ITRs) without estimating the ITEs by predicting the benefit under both treatments exist like the modified covariate method [28] or A-learning [12]. Additionally, approaches without explicitly relying on the ITE estimation can also be used like the constrained single-index regression [29] and many others [30–33]. However, it is unclear how to account for the heterogeneity that may arise between studies. To our knowledge, the only proposal of a non-regression-based approach to ITR development with IPD-MA data is a paper by Mistry et al. using recursive partitioning [34]. Investigating how to adapt those approaches that are less sensitive to model misspecification to incorporate an estimation of heterogeneity between studies should be studied in further work, for instance, building on approaches for federated learning [35, 36]. In our simulation settings, with a large sample size, and no complex interactions or non-linearities between variables, the regression models we used are expected to perform well, and there might be no clear advantage of more complicated approaches. But in more complex situations, this may not be the case, and these remain to be investigated as a follow-up of this work.

In this project, we chose to concentrate on ITE estimation using data from randomized control trials. In practice, personalized strategies have been developed using data from RCTs. The SYNTAX score II which aims to guide decision-making between coronary artery bypass graft surgery (CABG) and percutaneous coronary intervention (PCI) in patients with complex coronary artery disease was developed by Farooq et al. using data from the SYNTAX trial [2]. Other types of data could have been used to estimate ITEs such as observational data, provided that the confounding factors are accounted for. Most approaches for ITE estimation can be used with both RCTs and observational data. Another type of data that is often used in personalized medicine is genomics data. Some penalized methods that can be combined with the meta-learners used in this work have been proposed such as the group LASSO [19]. Studying how to correctly use the risk models that tackle heterogeneity with genomics data might be worth investigating.

We focused on estimating ITEs with a binary treatment. ITE estimation can also be done with multiple treatments. Extension of the meta-learners, that we used in this project, to multiple treatments have been done and compared in previous works [37, 38]. However, it is rare to find a meta-analysis of RCTs that all compare the same set of treatments in practice. The situation would likely be more relevant in the network meta-analysis (NMA) setting. To our knowledge, very few works have tackled the estimation of heterogeneous treatment effects in the NMA context [6]. How to handle heterogeneity between studies within a NMA framework for the ITE estimation may still warrant further study and developments.

IPD-MA benefits from a large sample size, which can facilitate the ITE estimation and can increase generalizability by including trials with not the exact same population. However, some challenges arise in using IPD-MA for estimating individualized treatment effects. Dealing with heterogeneity due to differences between trials is difficult and it was translated into poor discrimination and calibration in our case study. Another challenge, when a one-stage approach is used, is aggregation bias. Centering variables to their study-specific mean and including the covariate mean as an adjustment term can address this challenge [17].

Extensions of the present work could include the use of observational data instead of randomized control trial data. A further extension with observational data would be to develop methods to estimate this type of prediction models while allowing the datasets to remain located in different data warehouses, similar to the concept of federated learning [35, 36].

## Conclusion

In this paper, the performance of different strategies and methods dealing with heterogeneity between trials was evaluated to estimate the ITE with IPD-MA in a simulation study and in a clinical example. Results showed that, for the choice of the strategy, using interactions with treatment (S-learner) is preferable as it performs well with both binary and time-to-event outcomes and that, for the choice of the method, none of the methods we compared outperformed the other methods.

### Supplementary Information

**Supplementary Material 1.**

## Data Availability

The data that support the findings of this study are available from the INDANA collaboration but restrictions apply to the availability of these data, which were used under license for the current study, and so are not publicly available. Data are however available from the authors upon reasonable request and with permission of the INDANA collaboration. The code of the simulation study is available at https://github.com/floriebouvier/ITE-estimation-with-IPD-MA.

## Abbreviations

ITE: Individualized treatment effect
IPD-MA: Individual participant data meta-analysis
RI: Random intercept
SI: Stratified intercept
R1: Rank-1
FS: Fully stratified
SL: S-learner
TL: T-learner

## References

[1] Ballarini NM, Rosenkranz GK, Jaki T, Konig F, Posch M (2018). Subgroup identification in clinical trials via the predicted individual treatment effect. PLoS One..

[2] Farooq V, van Klaveren D, Steyerberg EW, Meliga E, Vergouwe Y, Chieffo A (2013). Anatomical and clinical characteristics to guide decision making between coronary artery bypass surgery and percutaneous coronary intervention for individual patients: development and validation of SYNTAX score II. Lancet..

[3] Debray TP, Moons KG, Ahmed I, Koffijberg H, Riley RD (2013). A framework for developing, implementing, and evaluating clinical prediction models in an individual participant data meta-analysis. Stat Med..

[4] Steyerberg EW, Nieboer D, Debray TPA, van Houwelingen HC (2019). Assessment of heterogeneity in an individual participant data meta-analysis of prediction models: An overview and illustration. Stat Med..

[5] Fisher DJ, Carpenter JR, Morris TP, Freeman SC, Tierney JF (2017). Meta-analytical methods to identify who benefits most from treatments: daft, deluded, or deft approach?. BMJ..

[6] Chalkou K, Steyerberg E, Egger M, Manca A, Pellegrini F, Salanti G. A two-stage prediction model for heterogeneous effects of many treatment options: application to drugs for Multiple Sclerosis. 2020. https://arxiv.org/pdf/2004.13464.pdf. Accessed 3 June 2021.

[7] Seo M, White IR, Furukawa TA, Imai H, Valgimigli M, Egger M (2020). Comparing methods for estimating patient-specific treatment effects in individual patient data meta-analysis. Stat Med..

[8] Künzel SR, Sekhon JS, Bickel PJ, Yu B (2019). Metalearners for estimating heterogeneous treatment effects using machine learning. Proc Natl Acad Sci U S A..

[9] Kennedy EH. Optimal doubly robust estimation of heterogeneous causal effects. arXiv. 2020. 10.48550/ARXIV.2004.14497.

[10] Susan Athey, Julie Tibshirani, Stefan Wager "Generalized random forests. Ann Stat Ann Statist. 2019;47(2):1148–78.

[11] Guo X, Ni A. Contrast weighted learning for robust optimal treatment rule estimation. Stat Med. 2022;sim.9574. 10.1002/sim.9574. 10.1002/sim.9574 PMC9826186 36104931

[12] Chen S, Tian L, Cai T, Yu M (2017). A general statistical framework for subgroup identification and comparative treatment scoring. Biometrics..

[13] Gueyffier F, Boutitie F, Boissel J, Coope J, Cutler J, Ekbom T (1995). INDANA: a meta-analysis on individual patient data in hypertension. Protocol and preliminary results. Thérapie..

[14] Royston P, Parmar MKB, Sylvester R (2004). Construction and validation of a prognostic model across several studies, with an application in superficial bladder cancer. Stat Med..

[15] Steyerberg EW, Moons KG, van der Windt DA, Hayden JA, Perel P, Schroter S (2013). Prognosis Research Strategy (PROGRESS) 3: prognostic model research. PLoS Med..

[16] van Klaveren D, Steyerberg EW, Serruys PW, Kent DM (2018). The proposed ‘concordance-statistic for benefit’ provided a useful metric when modeling heterogeneous treatment effects. J Clin Epidemiol..

[17] Riley RD, Debray TPA, Fisher D, Hattle M, Marlin N, Hoogland J (2020). Individual participant data meta-analysis to examine interactions between treatment effect and participant-level covariates: Statistical recommendations for conduct and planning. Stat Med..

[18] Belias M, Rovers MM, Reitsma JB, Debray TPA, IntHout J (2019). Statistical approaches to identify subgroups in meta-analysis of individual participant data: a simulation study. BMC Med Res Methodol..

[19] Lim M, Hastie T. Learning interactions through hierarchical group-lasso regularization. 2013. https://arxiv.org/abs/1308.2719. Accessed 3 June 2021. 10.1080/10618600.2014.938812 PMC4706754 26759522

[20] Pocock SJ, McCormack V, Gueyffier F, Boutitie F, Fagard RH, Boissel JP (2001). A score for predicting risk of death from cardiovascular disease in adults with raised blood pressure, based on individual patient data from randomised controlled trials. BMJ..

[21] Quartagno M, Carpenter JR (2016). Multiple imputation for IPD meta-analysis: allowing for heterogeneity and studies with missing covariates. Stat Med..

[22] White IR, Royston P, Wood AM (2011). Multiple imputation using chained equations: Issues and guidance for practice. Stat Med..

[23] Vergouwe Y, Royston P, Moons KGM, Altman DG (2010). Development and validation of a prediction model with missing predictor data: a practical approach. J Clin Epidemiol..

[24] Wood AM, Royston P, White IR (2015). The estimation and use of predictions for the assessment of model performance using large samples with multiply imputed data. Biom J..

[25] Imai K, Li ML (2021). Experimental Evaluation of Individualized Treatment Rules. J Am Stat Assoc..

[26] van Klaveren D, Balan TA, Steyerberg EW, Kent DM (2019). Models with interactions overestimated heterogeneity of treatment effects and were prone to treatment mistargeting. J Clin Epidemiol..

[27] Nie X, Wager S. Quasi-oracle estimation of heterogeneous treatment effects. Biometrika. 2020;asaa076. 10.1093/biomet/asaa076.

[28] Tian L, Alizadeh AA, Gentles AJ, Tibshirani R (2014). A Simple Method for Estimating Interactions Between a Treatment and a Large Number of Covariates. J Am Stat Assoc..

[29] Park H, Petkova E, Tarpey T, Ogden RT (2022). A Single-Index Model With a Surface-Link for Optimizing Individualized Dose Rules. J Comput Graph Stat..

[30] Zhang B, Tsiatis AA, Laber EB, Davidian M (2012). A robust method for estimating optimal treatment regimes. Biometrics..

[31] Zhao YQ, Zeng D, Laber EB, Song R, Yuan M, Kosorok MR (2015). Doubly Robust Learning for Estimating Individualized Treatment with Censored Data. Biometrika..

[32] Mo W, Qi Z, Liu Y (2021). Learning Optimal Distributionally Robust Individualized Treatment Rules. J Am Stat Assoc..

[33] Zhao YQ, Zeng D, Tangen CM, Leblanc ML (2019). Robustifying trial-derived optimal treatment rules for a target population. Electron J Stat..

[34] Mistry D, Stallard N, Underwood M (2018). A recursive partitioning approach for subgroup identification in individual patient data meta-analysis. Stat Med..

[35] Wu Y, Jiang X, Kim J, Ohno-Machado L (2012). Grid Binary LOgistic REgression (GLORE): building shared models without sharing data. J Am Med Inform Assoc..

[36] Lu CL, Wang S, Ji Z, Wu Y, Xiong L, Jiang X (2015). WebDISCO: a web service for distributed cox model learning without patient-level data sharing. J Am Med Inform Assoc..

[37] Zhao Y, Fang X, Simchi-Levi D. Uplift modeling with multiple treatments and general response types. 2017. http://arxiv.org/abs/1705.08492. Accessed 5 July 2023.

[38] Acharki N, Lugo R, Bertoncello A, Garnier J. Comparison of meta-learners for estimating multi-valued treatment heterogeneous effects. 2023. http://arxiv.org/abs/2205.14714. Accessed 11 Apr 2023.

