# Supplementary material for: Estimating individualized treatment effects using an individual participant data meta-analysis
Source: BMC Med Res Methodol. 2024 Mar 25;24:74. doi: 10.1186/s12874-024-02202-9 (PMC10962112; doi:10.1186/s12874-024-02202-9)
Supplement: Supplementary file 1 — Supplementary Material 1. [file 12874_2024_2202_MOESM1_ESM.pdf]

# Estimating individualized treatment effects using an individual participant data meta-analysis

Florie Bouvier Anna Chaimani Etienne Peyrot François Gueyffier
Guillaume Grenet Raphaël Porcher

## Supplementary material

## S1 Methods to estimate individualized treatment effects with a time-to-event outcome

### S1.1 ITE estimation

Let $S(t, x, z)$ represent the expected time-to-event at time $t$ under treatment $z$ for an individual with covariates $x$.
The ITE for a time-to-event outcome is estimated as:

$$\hat{\tau}(x) = \hat{S}(t, x, 1) - \hat{S}(t, x, 0)$$

at a prespecified time-point $t$.
Considering for instance a Cox regression model, the S-learner consists in estimating the following model:

$$S(t, x, z) = S_0(t) \exp(\theta' x + \gamma z).$$

From this, we derive for all individuals:

$$S(t, x, 1) = S_0(t) \exp(\theta' x + \gamma)$$

and

$$S(t, x, 0) = S_0(t) \exp(\theta' x).$$

With the T-learner, we fit the two following models:

$$S(t, x, 1) = S_0(t) \exp(\theta'^1 x + \gamma^1),$$

$$S(t, x, 0) = S_0(t) \exp(\theta'^0 x).$$

### S1.2 Risk prediction models

Using Cox regressions, the different models are expressed as:

- Naive model (NA):

$$\lambda_{ij}(t|x_{ij}) = \lambda_0(t) \exp(\theta x_{ij}) \quad (1)$$

  The individual predictions are obtained by $S(t, x) = \hat{S}_0(t) \exp(\theta x)$, where $\hat{S}_0(t) = \exp(-\hat{\Lambda}_0(t))$ and $\hat{\Lambda}_0(t) = \sum_{\tilde{t} \leq t} \hat{\lambda}_0(\tilde{t})$.

- Random intercept model (RI):

$$\lambda_{ij}(t|x_{ij}, \rho_j) = \lambda_0(t) \exp(\theta x_{ij} + \rho_j) \quad (2)$$

  where $\rho_j \sim \mathcal{N}(0, \tau_\rho^2)$. The individual predictions are obtained as $S(t, x|j) = \hat{S}_0(t) \exp(\theta x + \rho_j)$.

- Stratified intercept model (SI):

$$\lambda_{ij}(t|x_{ij}, \lambda_{0j}) = \lambda_{0j}(t)\exp(\theta x_{ij}) \tag{3}$$

The individual predictions are obtained as $S(t, x|j) = \hat{S}_{0j}(t)\exp(\theta x)$.

- Fully stratified (FS):

$$\lambda_{ij}(t|x_{ij}, \lambda_{0j}, \theta_j) = \lambda_{0j}(t)\exp(\theta_j x_{ij}) \tag{4}$$

The individual predictions are obtained as $S(t, x|j) = \hat{S}_{0j}(t)\exp(\theta_j x)$.

- Rank-1 (R1):

$$\lambda_{ij}(t|x_{ij}, \phi_j, \rho_j) = \lambda_0(t)\exp(\phi_j \theta x_{ij} + \rho_j) \tag{5}$$

The individual predictions are obtained as $S(t, x|j) = \hat{S}_0(t)\exp(\theta_x + \rho_j)$.

# S2 Potential aggregation bias

We did not simulate the fully stratified model. Since this model consists in stratifying every parameter by trial, aggregation bias is not an issue.

## S2.1 Simulations without ecological bias

We simulated a binary outcome following a Bernoulli distribution with parameter $P$ given by:

$$\text{logit}(P) = \beta_0 + \beta_1 x_1 + \beta_2 z + (\beta_3 x_1) \times z,$$

where $z$ denoted the binary treatment indicator, $x_1$ was a normally distributed variable (see parameterization in table S1). Values for the model parameters were: $\beta_0 = -1.4$, $\beta_1 = 0.02$, $\beta_2 = -0.3$ and $\beta_3 = 0.01$. A total of 1,000 simulations with an IPD-MA sample size of 2800 was performed, and models with and without variable centering as described in Riley et al. [1] were fitted to the data.

Table S1: Distribution of $x_1$.

| | Trial | | | | | | |
|---|---|---|---|---|---|---|---|
| Variable | 1 | 2 | 3 | 4 | 5 | 6 | 7 |
| $x_1$, $\mu$ ($\sigma$) | 52 (4) | 56 (2) | 64 (1) | 70 (3) | 77 (4) | 78 (6) | 82 (2) |

Table S2: Median parameter estimates (standard errors) over 1000 simulations with the S-learner when ecological bias was not included.

| | Model | Random intercept | | Stratified intercept | | Rank-1 | |
|---|---|---|---|---|---|---|---|
| Parameter | Value | No centering | Centering | No centering | Centering | No centering | Centering |
| $\beta_0$ | −1.4 | −1.40(0.07) | −1.40(0.10) | −1.27(0.33) | −1.42(0.86) | −1.40 | −1.41 |
| $\beta_1$ | 0.02 | 0.02(0.01) | 0.02(0.01) | 0.02(0.01) | 0.02(0.04) | 0.02 | 0.02 |
| $\beta_2$ | −0.3 | −0.30(0.11) | −0.31(0.11) | −0.30(0.11) | −0.31(0.11) | −0.30 | −0.30 |
| $\beta_3$ | 0.01 | 0.01(0.01) | −0.00(0.01) | 0.01(0.01) | 0.01(0.01) | 0.01 | 0.00 |

The parameters obtained with and without centering the variables were similar for all models(Table S2). The intercept obtained with the stratified intercept when centering was included, was closer to the real value than when centering was not included, however, its standard error was larger.

## S2.2 Simulations with ecological bias

We simulated a binary outcome in the same way as above but added some ecological bias. Therefore, the values for the model parameters were: $\beta_0 = -1.4$, $\beta_1 = 0.02$, $\beta_2 = -0.3 - ((mean(x_1) - 60)/100)$ and $\beta_3 = 0.01$.

Table S3: Median parameter estimates (standard errors) over 1000 simulations with the S-learner when ecological bias was included.

| | Model | Random intercept | | Stratified intercept | | Rank-1 | |
|---|---|---|---|---|---|---|---|
| Parameter | Value | No centering | Centering | No centering | Centering | No centering | Centering |
| $\beta_0$ | −1.4 | −1.40(0.08) | −1.40(0.10) | −1.22(0.34) | −1.42(0.88) | −1.34 | −1.43 |
| $\beta_1$ | 0.02 | 0.02(0.01) | 0.02(0.01) | 0.02(0.01) | 0.02(0.04) | 0.02 | 0.02 |
| $\beta_2$ | −0.40 | −0.40(0.12) | −0.40(0.12) | −0.40(0.12) | −0.40(0.12) | −0.40 | −0.40 |
| $\beta_3$ | 0.01 | 0.00(0.01) | −0.00(0.01) | −0.00(0.01) | −0.00(0.01) | 0.00 | 0.00 |

Centering or not centering the variables led to similar parameters (Table S3). Not centering the variables did not lead to aggregation bias, thus for simplicity, we decided to not add centering in this work.

# S3 Simulation settings

## S3.1 Covariates generation

In all simulation scenarios, covariates were numbered from $x_1$ to $x_3$ or $x_1$ to $x_9$, and their distribution varied among the trials $j$ of the meta-analysis, as detailed in the tables S4 and S5. Covariates were drawn either from Gaussian distribution with mean $\mu$ and standard deviation $\sigma$ or from a Bernoulli distribution with parameter $\pi$.

Table S4: Distribution parameters for covariates in scenarios with three covariates.

| | Trial | | | | | | |
|---|---|---|---|---|---|---|---|
| Variable | 1 | 2 | 3 | 4 | 5 | 6 | 7 |
| $x_1$, $\mu$ ($\sigma$) | 52 (4) | 56 (2) | 64 (1) | 70 (3) | 77 (4) | 78 (6) | 82 (2) |
| $x_2$, $\pi$ | 0.8 | 0.4 | 0.5 | 0.6 | 0.5 | 0.7 | 0.5 |
| $x_3$, $\mu$ ($\sigma$) | 186 (13) | 182 (16.5) | 170 (9.4) | 185 (12) | 190 (9) | 188 (10) | 197 (21) |

Table S5: Distribution parameters for covariates in scenarios with ten covariates.

| | Trial | | | | | | |
|---|---|---|---|---|---|---|---|
| Variable | 1 | 2 | 3 | 4 | 5 | 6 | 7 |
| $x_1$, $\mu$ ($\sigma$) | 52 (4) | 56 (2) | 64 (1) | 70 (3) | 77 (4) | 78 (6) | 82 (2) |
| $x_2$, $\pi$ | 0.8 | 0.4 | 0.5 | 0.6 | 0.5 | 0.7 | 0.5 |
| $x_3$, $\mu$ ($\sigma$) | 186 (13) | 182 (16.5) | 170 (9.4) | 185 (12) | 190 (9) | 188 (10) | 197 (21) |
| $x_4$, $\pi$ | 0.1 | 0.005 | 0.01 | 0.02 | 0.05 | 0.01 | 0.04 |
| $x_5$, $\pi$ | 0.002 | 0.06 | 0.02 | 0.02 | 0.001 | 0.008 | 0.04 |
| $x_6$, $\pi$ | 0.5 | 0.2 | 0.3 | 0.4 | 0.3 | 0.25 | 0.3 |
| $x_7$, $\pi$ | 0.03 | 0.001 | 0.002 | 0.07 | 0.003 | 0.01 | 0.002 |
| $x_8$, $\pi$ | 0.13 | 0.11 | 0.05 | 0.25 | 0.05 | 0.06 | 0.04 |
| $x_9$, $\mu$ ($\sigma$) | 176 (6) | 162 (9) | 167 (10) | 169 (10) | 168 (10) | 170 (9) | 167 (9) |

Table S6: Paramaters values for all scenarios.

| | No variation | | | | Variation | | | |
|---|---|---|---|---|---|---|---|---|
| | 3 covariates | | 9 covariates | | 3 covariates | | 9 covariates | |
| | Binary | TTE | Binary | TTE | Binary | TTE | Binary | TTE |
| $\beta_0$ | -1.4 | 50 | -1.4 | 50 | $-1.4 + ((mean(x_1[j]) - 60)/20)$ | $50 + ((mean(x_1[j]) - 60)/20)$ | $-1.4 + ((mean(x_1[j]) - 60)/20)$ | $50 + ((mean(x_1[j]) - 60)/20)$ |
| $\beta_1$ | 0.03 | 0.03 | 0.03 | 0.03 | 0.03 | 0.03 | 0.03 | 0.03 |
| $\beta_2$ | 0.7 | 0.7 | 0.7 | 0.7 | 0.7 | 0.7 | 0.7 | 0.7 |
| $\beta_3$ | 0.02 | 0.1 | 0.02 | 0.1 | $0.02 + 0.2 * prop(x_2[j])$ | $0.02 + 0.2 * prop(x_2[j])$ | $0.02 + 0.2 * prop(x_2)$ | $0.02 + 0.2 * prop(x_2[j])$ |
| $\beta_4$ | -0.3 | -0.3 | 0.82 | 0.82 | $-0.3 + ((mean(x_1[j]) - 60)/100)$ | $-0.3 + ((mean(x_1[j]) - 60)/100)$ | 0.82 | 0.82 |
| $\beta_5$ | 0.015 | 0.015 | 0.8 | 0.8 | 0.015 | 0.015 | 0.8 | 0.8 |
| $\beta_6$ | 0.1 | 0.1 | 0.7 | 0.7 | 0.1 | 0.1 | $0.7 + 0.2 * prop(x_2[j])$ | $0.7 + 0.2 * prop(x_2[j])$ |
| $\beta_7$ | -0.008 | -0.008 | 0.1 | 0.1 | -0.08 | -0.008 | $0.1 + ((mean(x_1[j]) - 60/100)$ | $0.1 + ((mean(x_1[j]) - 60/100)$ |
| $\beta_8$ | — | — | 0.33 | 0.33 | — | — | 0.33 | 0.33 |
| $\beta_9$ | — | — | -0.02 | -0.02 | — | — | -0.02 | -0.02 |
| $\beta_{10}$ | — | — | -0.3 | -0.3 | — | — | $-0.3 + ((mean(x_1[j]) - 60)/100)$ | $-0.3 + ((mean(x_1[j]) - 60)/100)$ |
| $\beta_{11}$ | — | — | 0.015 | 0.015 | — | — | 0.015 | 0.015 |
| $\beta_{12}$ | — | — | 0.04 | 0.04 | — | — | 0.04 | 0.04 |
| $\beta_{13}$ | — | — | 0.1 | 0.1 | — | — | 0.1 | 0.1 |
| $\beta_{14}$ | — | — | -0.008 | -0.008 | — | — | -0.008 | -0.008 |

### S3.2 Parameters generation

The binary outcome was simulated as:

$$\text{logit}(P) = \beta_0 + \beta_1 x_1 + \beta_2 x_2 + \beta_3 x_3 + \beta_4 z + (\beta_5 x_1 + \beta_6 x_2 + \beta_7 x_3) \times z$$

for scenarios with 3 covariates, and as:

$$\text{logit}(P) = \beta_0 + \beta_1 x_1 + \beta_2 x_2 + \beta_3 x_3 + \beta_4 x_4 + \beta_5 x_5 + \beta_6 x_6 + \beta_7 x_7 + \beta_8 x_8 + \beta_9 x_9 + \beta_1 0z + (\beta_1 1x_1 + \beta_1 2x_2 + \beta_1 3x_3 + \beta_1 4x_6) \times z$$

for scenarios with 9 covariates, where $z$ represents the binary treatment indicator.

The time-to-event outcome was simulated as:

$$\lambda(t) = \beta_0(t) exp(\beta_1 x_1 + \beta_2 x_2 + \beta_3 x_3 + \beta_4 z + (\beta_5 x_1 + \beta_6 x_2 + \beta_7 x_3) \times z)$$

for scenarios with 3 covariates, and as:

$$\lambda(t) = \beta_0(t) exp(\beta_1 x_1 + \beta_2 x_2 + \beta_3 x_3 + \beta_4 x_4 + \beta_5 x_5 + \beta_6 x_6 + \beta_7 x_7 + \beta_8 x_8 + \beta_9 x_9 + \beta_1 0z + (\beta_1 1x_1 + \beta_1 2x_2 + \beta_1 3x_3 + \beta_1 4x_6) \times z)$$

for scenarios with 9 covariates, where $z$ represents the binary treatment indicator.

### S3.3 Simulation scenarios

Table S7: Summary of the 24 simulation scenarios. IPD-MA: individual patients meta-analysis.

| Scenario | Outcome | No. covariates | IPD-MA sample size | Heterogeneity |
|---|---|---|---|---|
| 1 | Binary | 3 | 2800 | No |
| 2 | Binary | 3 | 1400 | No |
| 3 | Binary | 3 | 700 | No |
| 4 | Binary | 9 | 2800 | No |
| 5 | Binary | 9 | 1400 | No |
| 6 | Binary | 9 | 700 | No |
| 7 | Binary | 3 | 2800 | Yes |
| 8 | Binary | 3 | 1400 | Yes |
| 9 | Binary | 3 | 700 | Yes |
| 10 | Binary | 9 | 2800 | Yes |
| 11 | Binary | 9 | 1400 | Yes |
| 12 | Binary | 9 | 700 | Yes |
| 13 | Time-to-event | 3 | 2800 | No |
| 14 | Time-to-event | 3 | 1400 | No |
| 15 | Time-to-event | 3 | 700 | No |
| 16 | Time-to-event | 9 | 2800 | No |
| 17 | Time-to-event | 9 | 1400 | No |
| 18 | Time-to-event | 9 | 700 | No |
| 19 | Time-to-event | 3 | 2800 | Yes |
| 20 | Time-to-event | 3 | 1400 | Yes |
| 21 | Time-to-event | 3 | 700 | Yes |
| 22 | Time-to-event | 9 | 2800 | Yes |
| 23 | Time-to-event | 9 | 1400 | Yes |
| 24 | Time-to-event | 9 | 700 | Yes |

## S4 Simulation results

### S4.1 Scenarios 1 to 6 and 13 to 18

Figure S1 shows the results for scenarios 1 to 3 where the predictor effects did not vary across trials. The c-statistic for benefit values, without variation in the predictor effects, were similar between models. We

observed similar performances whether the S-learner or the T-learner was used. Looking at calibration, we found that the models' values are akin for both learners, the intercept values are close to the intended value of 0 but the slope values were far from 1. Concerning the MSE, choosing SI or R1 led to a higher MSE and therefore are not recommended. Increasing the size of the IPD-MA led to lower MSE values. Figure S2 represents the results for scenarios 4 to 6. FS led to worse performance than the other models with bad discrimination and calibration, as well as higher MSE values. The other models had a good performance with mean c-statistic for benefit values above 0.6 and good calibration results

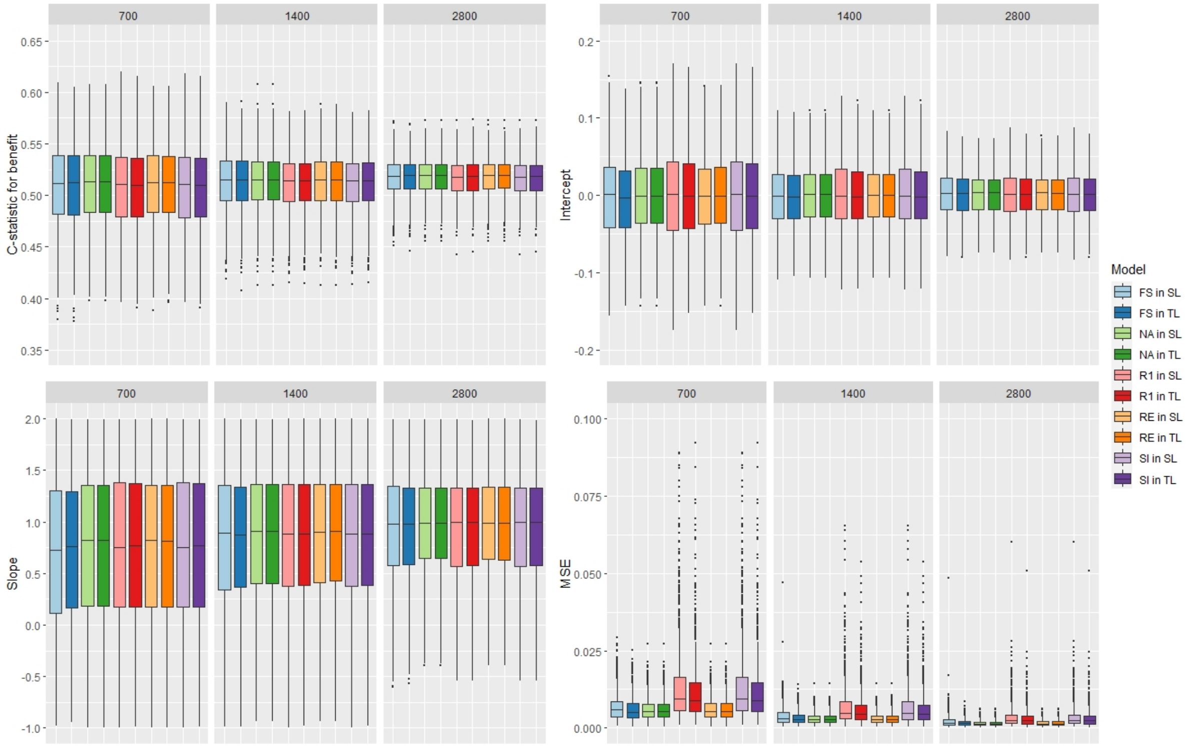


Figure S1: Boxplot of the measures of performance of the models for scenario 1 to 3.

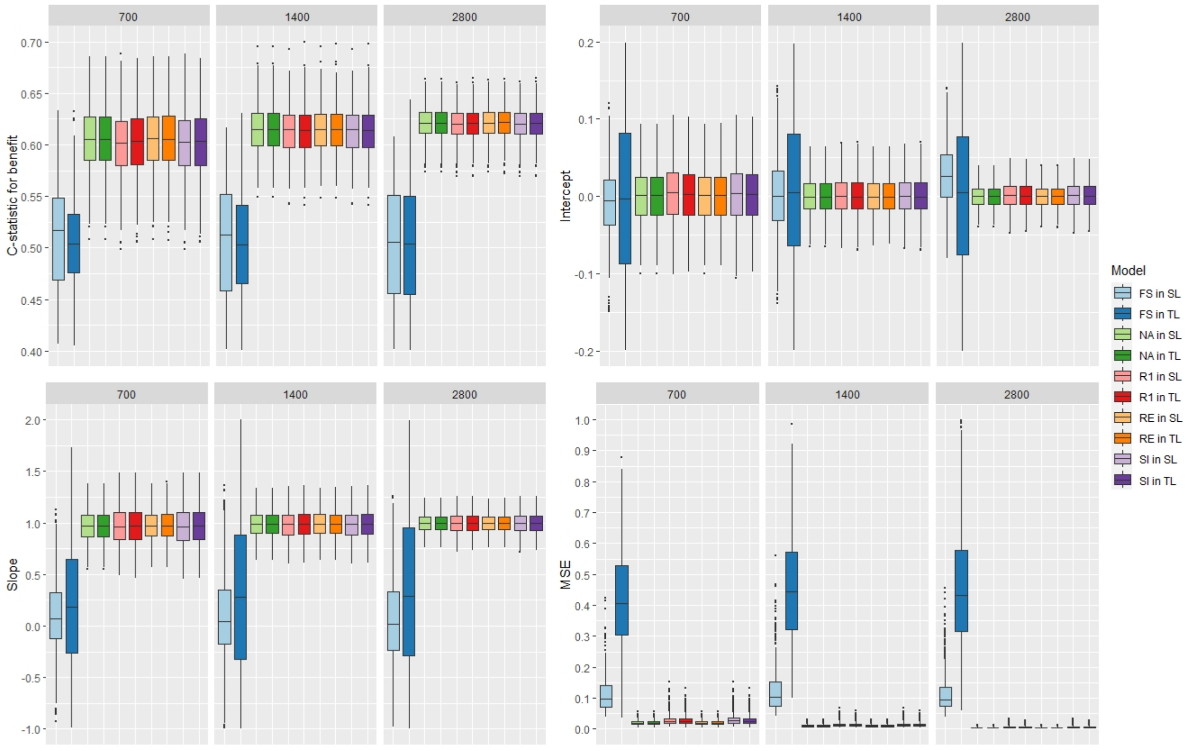


Figure S2: Boxplot of the measures of performance of the models for scenario 4 to 6.

The results of scenarios 13 to 15 are displayed in Figure S3. Using the T-learner led to better discrimination results for all methods, whereas using the S-learner led to better calibration results. Slope values far from 1 indicating a poor calibration. Higher MSE values were obtained for the fully stratified model and for the rank-1 model when the T-learner was used. The NA, RI, and SI methods' results were similar. Results of scenarios 16 to 18 are represented in Figure S5. FS had better calibration results when a time-to-event and 9 covariates were used. However, it had a lower c-statistic for benefit values and a higher MSE.

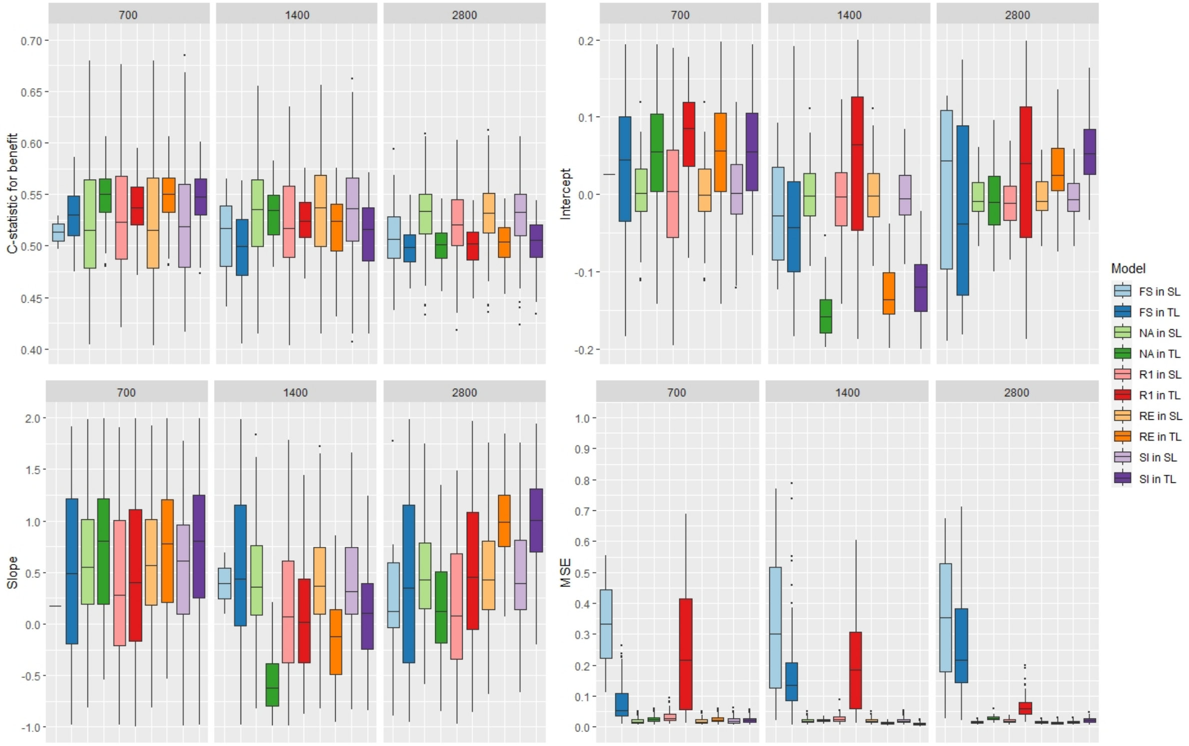


Figure S3: Boxplot of the measures of performance of the models for scenario 13 to 15.

Generally, the rank-1 and the fully stratified models which capture more heterogeneity in the predictor effects, were the two methods that captured more often the true ITE in their prediction intervals (Figure S4).

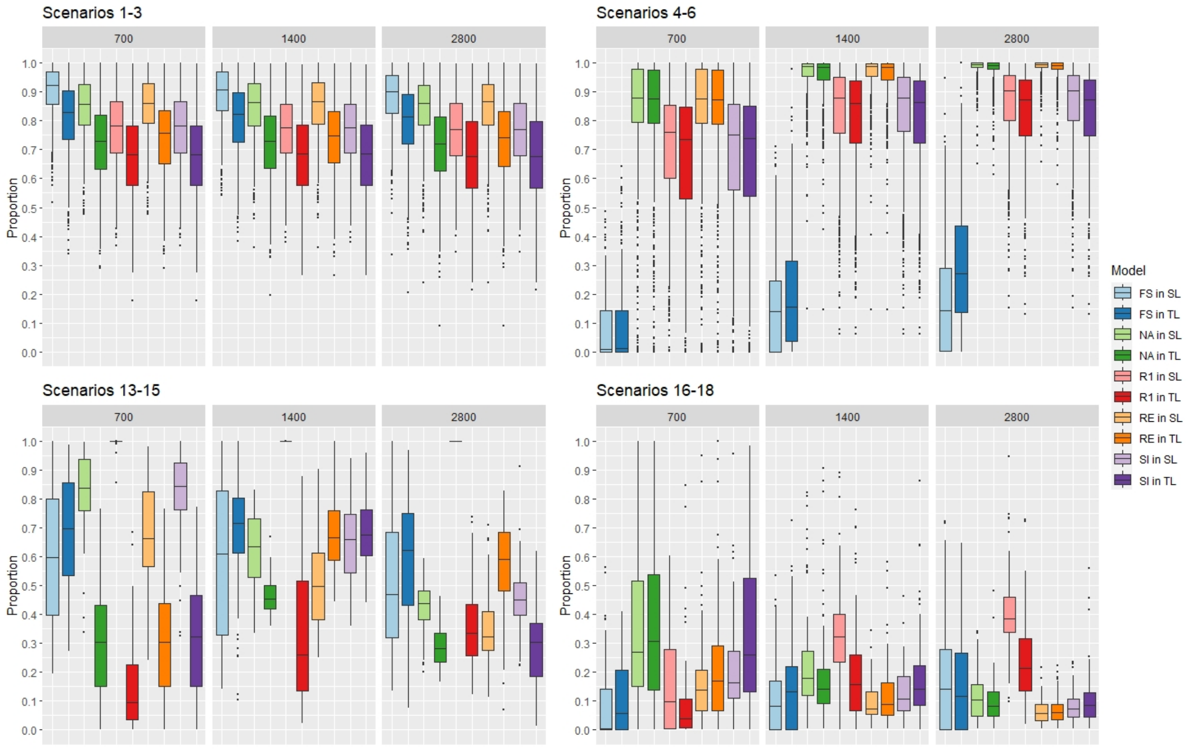


Figure S4: Number of times the true ITEs was in the prediction intervals of each model.

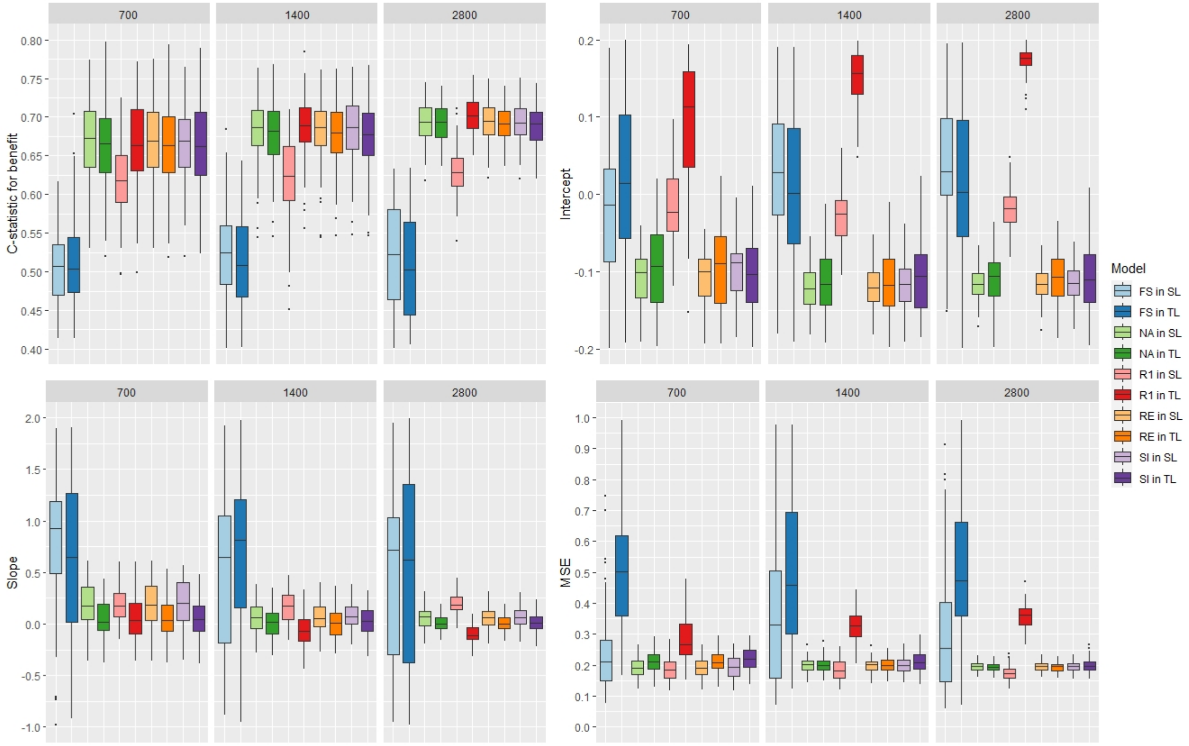


Figure S5: Boxplot of the measures of performance of the models for scenario 16 to 18.

Regarding the approaches with and without variables' selection using the stratified intercept method, the c-statistic for benefit values, intercept values and MSE values obtained were comparable whether variation in predictor effects was included or not (Figure S6 and Figure S7). Slope values differed a bit, but were of equivalent distance to the targeted value of 1. These observations were the same whether the method was used with the S-learner or the T-learner approach. When variation was included, slope values were slightly closer to 1 with variable selection and the T-learner approach. Adding variables' selection did not significantly impact the performances and did not modify our conclusions.

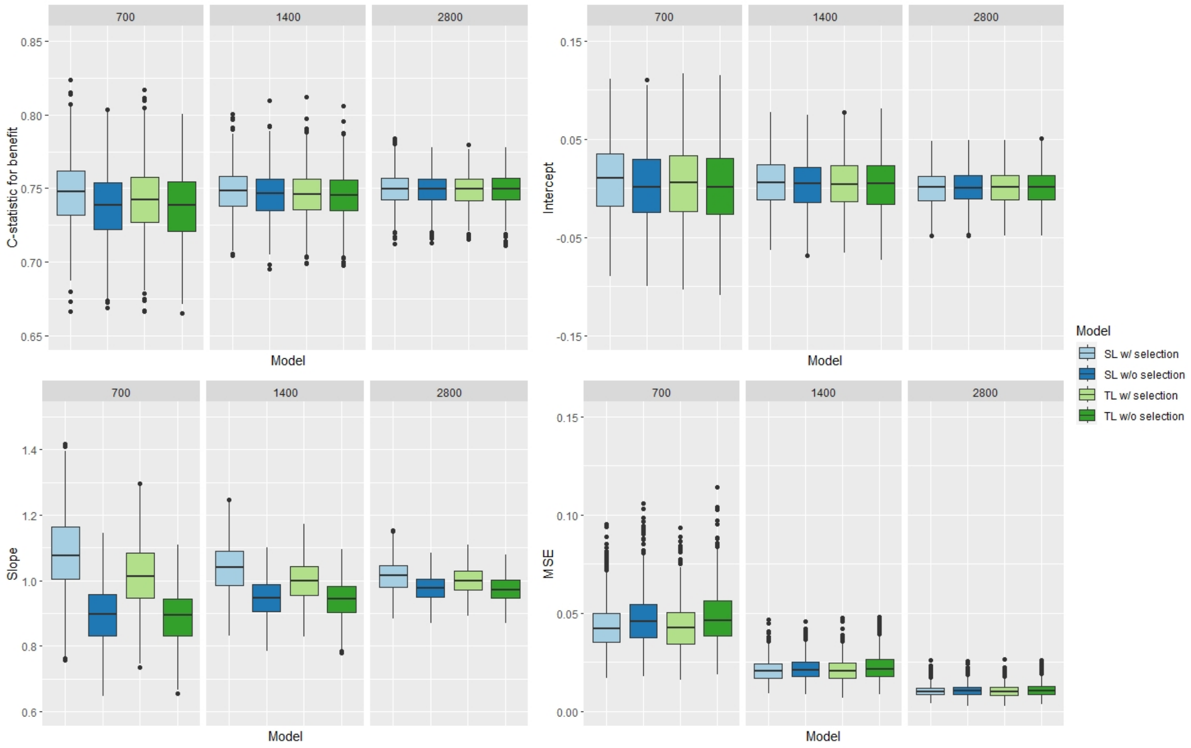


Figure S6: Boxplot of the measures of performance of the models for scenario 4 to 6 with and without variables' selection.

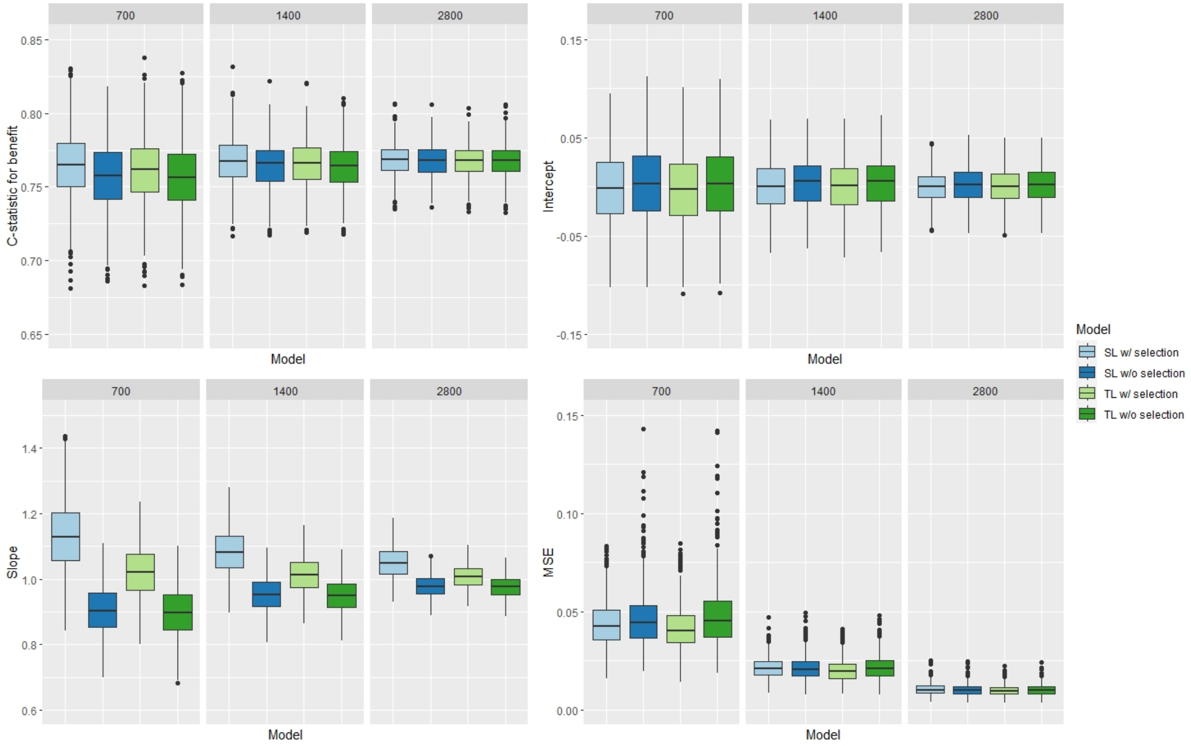


Figure S7: Boxplot of the measures of performance of the models for scenario 10 to 12 with and without variables' selection.

## S4.2 Scenario with a proportional heterogeneity

We took scenario 7 but changed the heterogeneity of the treatment variable ($\beta_4$) and variable $\beta_3$, described in Table 6. In this new scenario, the heterogeneity of $\beta_3$ is generated as $\{0.02, -0.02, 0.02, 0.04, 0.01, -0.04, 0.03\}$ and the heterogeneity of $\beta_4$ is generated as $\{-0.05, -0.025, 0.025, 0.025, 0.015, 0.05, 0.1\}$.
We obtained better calibration results and a slightly higher median number of times the true ITE was in the prediction interval (Table S8).

Table S8: Median results of the rank-1 model for a scenario in which the heterogeneity was generated in a proportional way.

| | S-learner | T-learner |
|---|---|---|
| C-statistic for benefit | 0.531 | 0.531 |
| Calibration's intercept | 0.001 | 0.004 |
| Calibration's slope | 1.074 | 1.071 |
| MSE | 0.002 | 0.002 |
| Proportion in prediction interval | 0.870 | 0.799 |

# S5 INDANA IPD-MA

## S5.1 Distribution of age

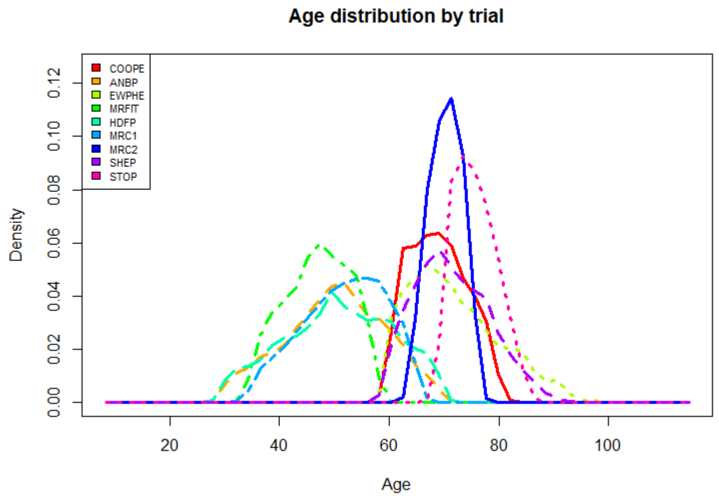


Figure S8: Distribution of age in each trial of INDANA.

## S5.2 Results on train dataset

When assessing the performance of the methods and the approaches on the train dataset, the discrimination remained low but the calibration improved, especially for the SI and R1 methods (Table S9). The fact that the performance did not increase a lot on the train dataset might indicate that the disparity between the trials was too high for them to be meta-analyzed.

Table S9: Median results using INDANA with a binary outcome with the training dataset.

| | S-learner | | | | | T-learner | | | | |
|---|---|---|---|---|---|---|---|---|---|---|
| | NA | RI | SI | R1 | FS | NA | RI | SI | R1 | FS |
| C-stat | 0.534 | 0.532 | 0.531 | 0.531 | 0.532 | 0.517 | 0.528 | 0.529 | 0.529 | 0.516 |
| Intercept | -0.001 | 0.001 | 0.001 | 0.001 | 0.001 | -0.001 | -0.002 | -0.001 | -0.001 | -0.002 |
| Slope | 1.376 | 1.183 | 1.133 | 1.133 | 0.954 | 0.868 | 0.905 | 0.984 | 0.984 | -0.063 |
| MSE | 0.000 | 0.000 | 0.000 | 0.000 | 0.000 | 0.000 | 0.000 | 0.000 | 0.000 | 0.000 |

# References

[1] R. D. Riley, T. P. A. Debray, D. Fisher, M. Hattle, N. Marlin, J. Hoogland, F. Gueyffier, J. A. Staessen, J. Wang, K. G. M. Moons, J. B. Reitsma, and J. Ensor. Individual participant data meta-analysis to examine interactions between treatment effect and participant-level covariates: Statistical recommendations for conduct and planning. *Statistics in Medicine*, 39(15):2115–2137, 2020.
